# Anion Defects Engineering of Ternary Nb-Based Chalcogenide Anodes Toward High-Performance Sodium-Based Dual-Ion Batteries

**DOI:** 10.1007/s40820-023-01070-0

**Published:** 2023-04-15

**Authors:** Yangjie Liu, Min Qiu, Xiang Hu, Jun Yuan, Weilu Liao, Liangmei Sheng, Yuhua Chen, Yongmin Wu, Hongbing Zhan, Zhenhai Wen

**Affiliations:** 1 College of Materials Science and Engineering, Fuzhou University, Fuzhou, 350108 People’s Republic of China; 2 CAS Key Laboratory of Design and Assembly of Functional Nanostructures, and Fujian Provincial Key Laboratory of Materials and Techniques Toward Hydrogen Energy, Fujian Institute of Research on the Structure of Matter, Chinese Academy of Sciences, Fuzhou, Fujian 350002 People’s Republic of China; 3 Fujian Normal University, Fuzhou, 350108 People’s Republic of China; 4 State Key Laboratory of Space Power-Sources Technology, Shanghai Institute of Space Power Sources, 2965 Dongchuan Road, Shanghai, 200245 People’s Republic of China

**Keywords:** NbSSe, Sodium-based dual-ion battery, Anode, Nanosheets architecture, Anion defects engineering

## Abstract

**Highlights:**

- We developed an efficient and extensible strategy to produce the single-phase ternary NbSSe nanohybrids with defect-enrich microstructure.
- The anionic-Se doping play a key role in effectively modulating the electronic structure and surface chemistry of NbS_2_ phase, including the increased interlayers distance (0.65 nm), the enhanced intrinsic electrical conductivity (3.23 × 10^3^ S m^-1^) and extra electroactive defect sites.
- The NbSSe/NC composite as anode exhibits rapid Na+ diffusion kinetics and increased capacitance behavior for Na^+^ storage, resulting in high reversible capacity and excellent cycling stability.

**Abstract:**

Sodium-based dual-ion batteries (SDIBs) have gained tremendous attention due to their virtues of high operating voltage and low cost, yet it remains a tough challenge for the development of ideal anode material of SDIBs featuring with high kinetics and long durability. Herein, we report the design and fabrication of N-doped carbon film-modified niobium sulfur–selenium (NbSSe/NC) nanosheets architecture, which holds favorable merits for Na^+^ storage of enlarged interlayer space, improved electrical conductivity, as well as enhanced reaction reversibility, endowing it with high capacity, high-rate capability and high cycling stability. The combined electrochemical studies with density functional theory calculation reveal that the enriched defects in such nanosheets architecture can benefit for facilitating charge transfer and Na^+^ adsorption to speed the electrochemical kinetics. The NbSSe/NC composites are studied as the anode of a full SDIBs by pairing the expanded graphite as cathode, which shows an impressively cyclic durability with negligible capacity attenuation over 1000 cycles at 0.5 A g^−1^, as well as an outstanding energy density of 230.6 Wh kg^−1^ based on the total mass of anode and cathode.
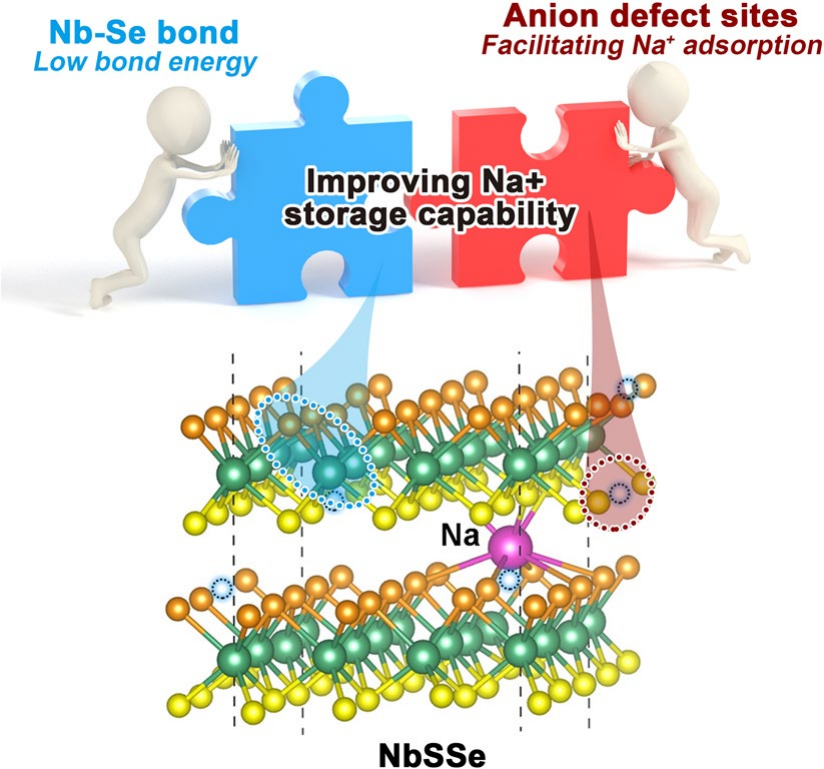

**Supplementary Information:**

The online version contains supplementary material available at 10.1007/s40820-023-01070-0.

## Introduction

Sodium-based dual-ion batteries (SDIBs) have become emerging techniques with potential application in the fields of energy storage thanks to their various advantages, such as high output voltage and the rich sodium resource, as well as environmental compatibility of nontransition-metal cathode [1, 2]. However, the larger size of Na^+^ (0.102 nm vs. Li^+^ 0.076 nm) leads to slow kinetics of ion transport and huge volumetric variation of anode during sodiation/desodiation process [3]. Graphite, as the commercial anodes of lithium-ion battery, yet is not fit for Na^+^ storage due to the low reversible Na^+^ storage capacity (around 30 mAh g^−1^ for NaC_64_) [4, 5]. Therefore, it is vitally important to explore appropriate materials to meet the requirement of Na^+^ storage.

As a typical transition-metal chalcogenide, niobium disulfide (NbS_2_) has been regarded as a prospective candidate as it has a well-defined layered structure with large lattice spacing (0.62 nm), high theoretical specific capacity (683 mAh g^−1^) and a rather preferable electrical conductivity (1 × 10^3^ S m^−1^) [6]. However, the bulk NbS_2_ for Na^+^ storage faces several key challenges, including the poor accessibility of active sites and the slow inferior electron/ion conductivity, which has a limited capacity and sluggish kinetic [7]. In addition, NbS_2_ tends to agglomerate and pulverize, and the severe volume variation upon the de-/sodiation process that finally results in rapid capacity degradation and inferior cycling capability. To settle these issues, some researchers have reported that the optimization of component and microstructure can effectively enhance electrochemical performance of anode [8]. Although appreciable progress has been made, the conversion reaction kinetics of NbS_2_ electrode is still restricted by the intrinsic high reaction energy barriers due to high bond dissociation energy barrier of Nb–S bonds (> 320 kJ mol^−1^) [9, 10].

Recently, the anion defect engineering has been regarded as an effective route to improve the electrochemical activity of nanomaterials upon discharging/charging process: (1) The electrons in the defect sites can be stimulated into the conduction band, producing a narrowing of the bandgap which is conducive to enhancing the charge transfer dynamics; (2) moreover, the anion defects would modify the local electronic properties of adjacent atoms, thus leading to reduce the decomposition activation energy of intermediate and improve the efficiency of conversion reaction [11, 12]. Selenium (Se) presents a similar physicochemical character to sulfur (S), while it has a larger atomic size and lower electronegativity. Thus, the in situ doping of Se into the lattice of NbS_2_ should be a viable route to create anion defect sites and to improve the conductivity [13, 14]. In addition, the relative low bond dissociation energy of Nb–Se to Nb–S favors the reversible conversion reaction for improving the reversibility, which benefits for enhancing the capability to store Na^+^ and expediting the reaction kinetics [4, 15]. However, the incorporation of anion defects into the NbS_2_ anode has never been reported; and also, the scientific understanding of the defect structure–performance relationship on Na^+^ storage behavior needs to be further in-depth studied.

Herein, we report the synthesis of single-phase NbSSe nanoarchitecture hybrids (NbSSe/NC) with N-doped carbon film decorating few-layer sheets as building blocks. Such NbSSe/NC nanosheets architecture with S–Nb–Se interlayer ligands combines the desired merits of few-layered structure with expanded interlayer space, improved electrical conductivity and defect-enriched microstructure. Accordingly, the NbSSe/NC nanohybrids exhibit highly impressive electrochemical properties toward sodium-ion storage with high specific capacity and long-term durability, which prompt us to develop a high-performance sodium-based dual-ion battery with high energy density and long-term durability.

## Experimental Section

### Chemicals

All the reagents were directly used without further purification. Niobium chloride (NbCl_5_, 99.9%) and selenium powder (Se, ≥ 99.99% metals basis) were purchased from Shanghai Macklin Biochemical Co., Ltd.; 1-octadecene (ODE, 90%) and oleylamine (OA, 98%) were purchased from Adamas-beta; and carbon disulfide (CS_2_, Aldrich, 99.9%) was from Sinopharm Chemical Reagent Co., Ltd. All chemicals were used without further purification.

### Synthesis

#### Synthesis of NbS_2_–OA

First, 250 mg NbCl_5_, 10 mL of OA and 10 mL ODE were mixed in a three-neck flask. The mixture was degassed under a vacuum for 20 min at room temperature to remove H_2_O and O_2_ and then heated to 140 °C for 30 min under argon atmosphere protection. Subsequently, the solution was further heated 280 °C with a heating rate of 5 °C min^−1^. While heating, the orange color was turned to light brown. Once the system reached 280 °C, 3 mL CS_2_ solution was slowly injected into the flask. After injection, the reaction temperature was maintained at 280 °C for 60 min. After that, the brown precipitates (NbS_2_–OA) were collected by centrifugation, washed with *n*-hexane and methanol for several times and vacuum-dried at 70 °C for one night. The NbS_2_ was obtained in the same way as NbS_2_–OA without adding the OA.

#### Synthesis of NbSSe/NC, NbS_2_/NC and NbS_2_

To prepare the NbSSe/NC composites, the NbS_2_–OA precursor and selenium powder were mixed with a molar ratio of S:Se (1:1), and then, the mixture was heated at 500 °C for 2 h in a sealed glass bottle under H_2_/Ar protection. For comparison, the NbS_2_/NC composite was prepared in the same way as NbSSe/NC, just without selenium powder; the NbS_2_ composite was prepared in the same way as NbS_2_/NC, just without OA.

### Characterization

The morphologies of materials were characterized by FESEM (Hitachi SU-8020) and HRTEM (Tecnai F20). The structure and composition of products were measured by XPS (ESCALAB 250Xi, Thermo Fisher), XRD (Miniflex 600 powder X-ray diffractometer with Cu Kα radiation in the 2θ range from 10° to 70°) and Raman spectrum (LabRam HR800). The electronic conductivity was tested by a four-point probe method at the current of 0.02 mA (RTS-8, 4Probes Tech Ltd.). EPR spectra were recorded using a Bruker spectrometer (ELEXSYS E500).

The specific surface area was performed using the Brunauer–Emmett–Teller equation by nitrogen adsorption and desorption isotherm (IGA100B). TGA test of the samples was performed by a simultaneous thermal analyzer (STA 449 F3 Jupiter, NETZSCH).

### Electrochemical Measurements

#### Anode and Cathode Preparation

The negative electrodes consisted of the 80 wt% active materials (NbSSe/NC, NbS_2_/NC, or NbS_2_), 10 wt% Ketjen Black and 10 wt% carboxyl methyl cellulose (CMC) binder onto Cu foil. The loading mass of anode was maintained at about 1.2 ~ 1.5 mg cm^−2^. The electrochemical measurements were investigated with 2032-type coin cells assembled in a glove box filled with argon atmosphere (< 0.5 ppm of H_2_O and O_2_).

#### For the Half-cell

The metallic Na foil was used as the counter and reference electrodes, the glass fiber (Whatman) as the separator film and 1 M NaPF_6_ dissolved in ethylene carbonate/ethyl methyl carbonate/dimethyl carbonate (EC/EMC/DMC, 1:1:1, v/v/v), and 7% FEC was used as the electrolyte. The electrolyte/active material ratio in each cell was ~ 100 μL mg^−1^. The specific capacity and specific currents were calculated based on the weight of anode materials.

#### For the Sodium-based Dual-ion Full Cell

The preparation method of EG cathode was same with anode, just employing the polyvinylidene fluoride (PVDF) as binder and Al foils as current collector. The areal loading of EG cathode was about 5 ~ 6 mg cm^−2^. The mass ratio of anode to cathode was preset to about 1:4. The electrolyte was the 3 M NaPF_6_ in the solution of EC/EMC/DMC and 7% FEC. The cyclic voltammetry (CV) curves were measured by a CHI660E electrochemical workstation. The galvanostatic charge/discharge (GCD) cycling and rate capability tests were conducted on a Neware battery test system (CT-ZWJ-4’S-T-1U, Shenzhen, China) with the voltage range of 0.01–3 V for SIBs and 1.5–4.8 V for SDIBs. For the long-term cycling performance at high rate, the SDIBs were first activated at 0.1 A g^−1^ for ten cycles and then were operated at a higher specific current for a long cycle test. An ac voltage amplitude of 5.0 mV was employed to measure EIS within the frequency range from 10 mHz to 100 kHz.

### Calculation Method

The DFT calculations were performed with periodic super-cells under the generalized gradient approximation (GGA) using the Perdew–Burke–Ernzerhof (PBE) function for exchange–correlation and the ultrasoft pseudopotentials for nuclei and core electrons. The Kohn–Sham orbitals were expanded in a plane-wave basis set with a kinetic energy cutoff of 30 Ry and the charge density cutoff of 300 Ry. The adsorption energy ΔE_a_ can be calculated by Eq. 1:

1 $$ \Delta E_{a} = E_{{{\text{tot}}}} - E_{{{\text{Na}}}} - E_{{{\text{str}}}} $$

where *E*_tot_ is the total energy of compound obtained from DFT calculations, *E*_Na_ is the energy of Na atoms and *E*_str_ is the energy of each structure. Electron density difference was calculated by subtracting the charge densities of Na atom and each configuration from the corresponding compounds. The charge density difference can be used to analyze the bonding process or the charge transfer before and after structural relaxation. The charge density difference of system can be calculated by Eq. 2:

2 $$ \Delta \rho = \rho_{{{\text{AB}}}} - \rho_{A} - \rho_{B} $$

where ρ_AB_ is the composition, ρ_A_ is the base and ρ_B_ is the absorbate. In calculation of the latter two quantities, the atomic positions are fixed as those they have in the AB system. The Fermi surface effects have been treated by the smearing technique of Methfessel and Paxton, using a smearing parameter of 0.02 Ry. The Brillouin zones were sampled with a k-point with gamma point. A supercell of 3 × 3 × 1 is adopted. The diffusion barrier for Na atom was determined by nudged elastic band (NEB). All the DFT calculations are implemented by the PW and NEB modules contained in the Quantum ESPRESSO distribution.

## Results and Discussion

### DFT Analysis of the Effect of Se Dopants on Na^+^ Storage

**Fig. 1:**
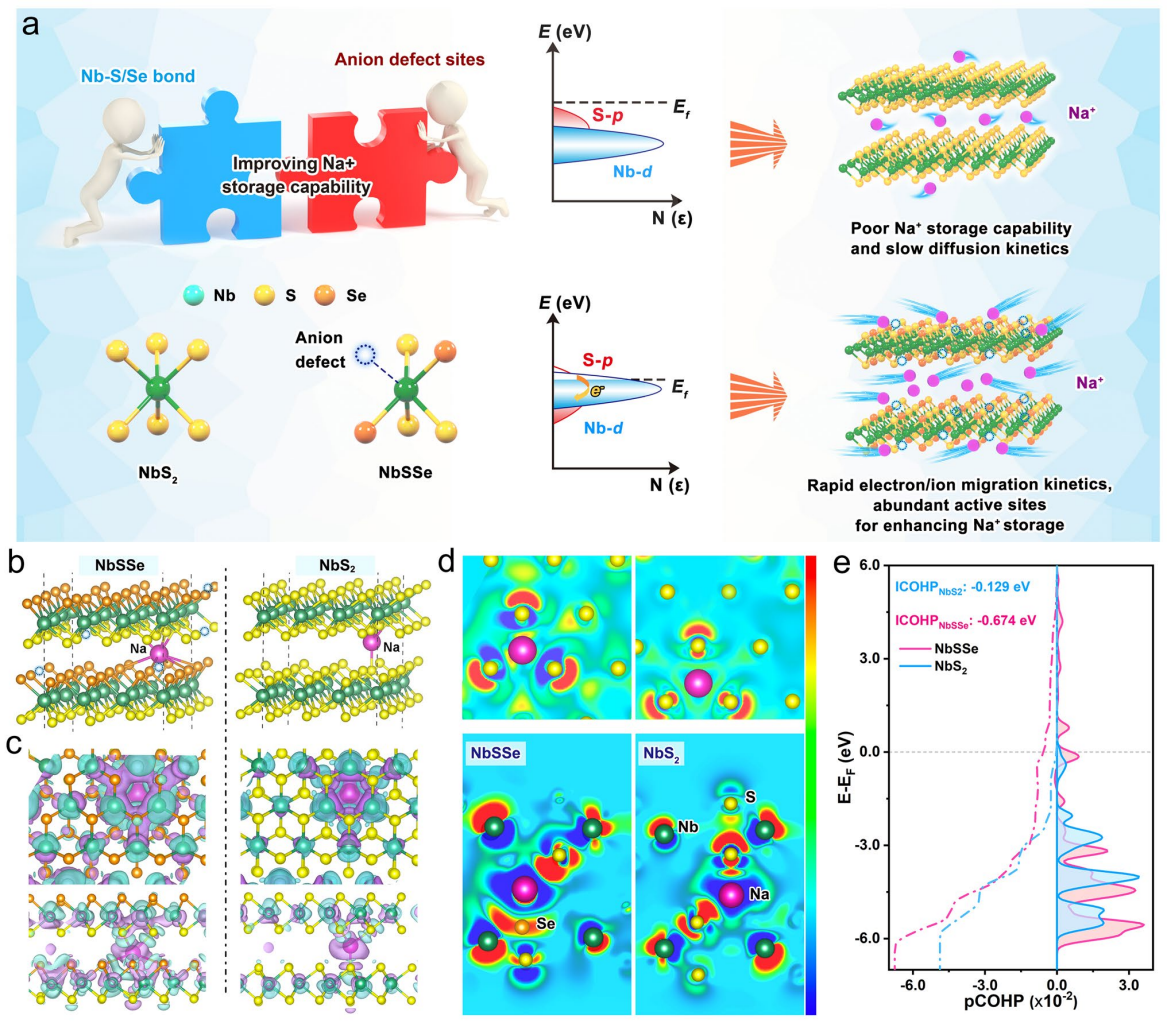
**a** Schematic illustration of charge self-regulation effect on manipulating active electronic states. **b** The optimized structures of NbSSe and NbS_2_ for Na^+^ adsorption. **c** 3D electron density difference distribution and **d** cross-sectional diagram of intercalated Na in NbS_2_ and NbSSe. **e** Average pCOHP and the corresponding integral patterns for NbS_2_ and NbSSe

Figure 1a presents the difference of the basal plane activation in active electronic states between NbS_2_ and NbSSe. The lower d-band center in NbS_2_ leads to weak chemical reactivity with Na^+^. The introducing of Se atoms into NbS_2_ crystal lattice favors to introduce a charge self-regulation effect because the activated d orbital of Nb and p orbital of S across the Fermi surface in NbSSe structure, effectively accelerating the electron transfer during the electrochemical process. In addition, the generation of anion defects would provide rich active defect sites for contributing the surface pseudocapacitive capacity for Na^+^ storage and fast kinetics.

Furthermore, the first-principles calculations were done to study the structural advantage of NbSSe for Na^+^ storage. The optimized computational models with Se doping are depicted in Figs. 1b and S1–S2. The computation of average adsorption energy (Δ*E*_a_) demonstrates that defect-enriched NbSSe structure displays stronger adsorption of Na ions by lower adsorption energy (− 2.29, − 3.98 and − 5.64 eV), whereas the corresponding values for NbS_2_ are − 1.64, − 3.14 and − 4.67 eV, which implies the defect sites would provide effective adsorption for the increased surface-induced pseudocapacitive charge storage (Fig. S3) [16]. Figure 1c presents the electron density difference models for the adsorption of Na atoms on the surface of samples, while Fig. 1d is a slice of Fig. 1c. They show that defect-rich structured NbSSe would induce obvious interfacial charge redistribution compared to the defect-free NbS_2_, indicative of a stronger interaction between Na and surface materials, leading to higher redox reaction activity [17]. The crystal orbital Hamilton population (COHP) analysis is performed to study the interaction of Nb–S bond in NbS_2_ and NbSSe (Fig. 1e). We found the integrated COHP (ICHOP, − 0.674 eV) value of NbS_2_ is significantly smaller than that of NbSSe (−0.129 eV) near the Fermi level, which implies the introduction of selenium ions would make Nb–S bond easier to trigger the redox reaction, resulting in more kinetically favorable and higher electrochemical reversibility [18]. Therefore, the coupling of the anion defect construction and charge self-regulation effect can availably improve Na^+^ adsorption and migration ability, therefore theoretically verifying the enhanced Na^+^ storage property.

### Synthesis and Characterizations of the NbSSe/NC

**Fig. 2:**
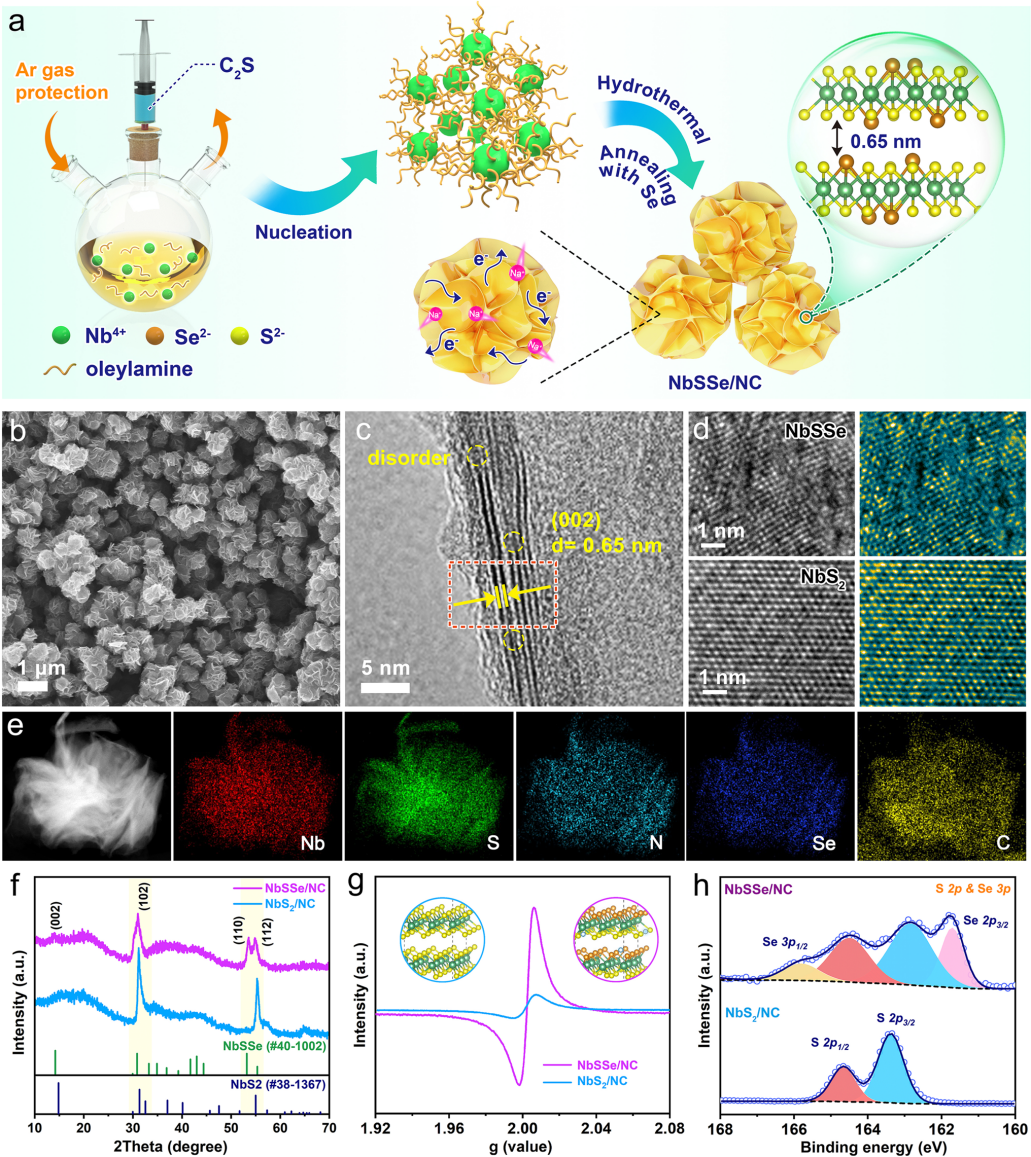
**a** Schematic strategy of synthesis for the NbSSe/NC composites. **b** SEM images and **c** HRTEM images of NbSSe/NC, **d** atomic resolution pictures of NbSSe/NC and NbS_2_/NC composites and related FFT-filtered atomic resolution images, **e** elemental mapping images of NbSSe/NC, **f** XRD pattern, **g** EPR results, **h** S *2p* and Se *3p* high-resolution XPS spectrum of NbSSe/NC and NbS_2_/NC

For the purpose of demonstrating our concept, the Se-doped NbS_2_ nanohybrid electrode is prepared, as schematically illustrated in Fig. 2a. NbCl_5_ as the precursor was added into the hot bath composed of oleylamine (OA) and 1-octadecene (ODE) under an argon atmosphere to form the coordination complex of niobium chloro oleylamine (NbCl–OA) [19]; the mixture was then heated to 280℃ followed by slow injection of CS_2_ for assembling into OA-modified NbS_2_ nanospheres (NbS_2_–OA) (Fig. S4), which was finally evolved into NbSSe composite through selenization process upon annealing, during which the OA was carbonized into N-doped carbon layer (denoted as NbSSe/NC).

Figure 2b exhibits field-emission scanning electron microscopy (FESEM) images of the NbSSe/NC nanohybrids, which display the nanoflower-like architecture with an average size of 800 ~ 900 nm with numerous two-dimensional building blocks of nanosheets. The transmission electron microscopy (TEM) and high-resolution TEM were conducted to study the fine structure. As shown in Figs. 2c and S5, one can observe that the NbSSe nanosheets are in the few-layer structure (2–4 layers), showing an expended interlayer distance of 0.65 nm, which is larger than the (002) crystalline planes that of NbS_2_ (0.61 nm). For comparison, the NbS_2_/NC composite was also prepared by direct calcination of the NbS_2_–OA precursor, which displayed a similar structure with NbSSe (Fig. S6), while for pure NbS_2_, their nanosheets tended to agglomerate and failed to form nanoflower morphology (Fig. S7). More importantly, as shown in Fig. 2d, the atomic resolution images and the related fast Fourier transformation (FFT)-filtered pictures were utilized to visualize the NbSSe/NC and NbS_2_/NC composites at a sub-angstrom resolution, which suggested obvious internal defects in the NbSSe basal plane; while for NbS_2_/NC, a ordered hexagonal atomic lattice can be observed. The energy-dispersive spectroscopy (EDS) and corresponding mapping images showed uniform distribution of Nb, S, Se, C and N elements with the Se/S atomic ratio of ~ 1:1 (Figs. 2e and S8). These results indicate the Se atoms doping in NbS_2_ lattice can effectively enlarge interlayer spacing and generate rich defects.

Figure 2f displays the X-ray diffraction (XRD) patterns of the NbSSe/NC; all characteristic peaks can be well indexed to NbSSe phase (JCPDS #40-1002), which is similar to the hexagonal crystal structure of NbS_2_ (JCPDS No. 38-1367) [7]. Notably, the (002), (102) and (110) planes of the NbSSe/NC present an obvious negative shift to lower degree, implying an enlarged interlayer space induced by the introduction of large-sized Se atoms into NbS_2_, which is in accordance with the TEM observation. To further confirm the phase evolution from NbS_2_ to NbSSe, the Raman measurement was further carried out (Fig. S9). Two distinct characteristic peaks detected at ~ 370.2 and ~ 440.3 cm^−1^ in the NbS_2_ sample are attributed to the in-plane E_2g_ vibration and out-plane A_1g_ vibration modes of Nb–S band, respectively [20]. As for the NbSSe/NC sample, both Nb–S modes shift negatively to the low-frequency range compared with NbS_2_/NC, indicating the decreased symmetry of Nb–S bonds due to Se doping, and the formation of Nb–Se bond can be detected by the Nb–Se vibration at a wave number of 312.9 cm^−1^ [21]. The inductively coupled plasma result (ICP) reveals the elemental ratio of Nb:S is 1:1.95 for NbS_2_/NC sample (Table S1), which is very close to the expected stoichiometry value. The elemental ratio among Nb, S and Se in NbSSe/NC is 1:0.9:0.9, which indicates the successful substitution of S with Se. The defects structure was further studied by electron paramagnetic response (EPR), as shown in Fig. 2g. The NbSSe/NC exhibits a couple of conspicuous peaks near a *g*-factor of 2.003, which almost show one order magnitude higher than that of NbS_2_/NC. This indicates the anion substitution of S by Se can generate abundant structure defects in NbS_2_ lattice, which endows electrode materials with rich active sites and improved electrical conductivity, consequently conducting to the rapid charge carrier transport.

X-ray photoelectron spectroscopy (XPS) spectra were measured and utilized for analyzing the surface states of NbS_2_-based nanomaterials. The survey XPS spectrum indicates the coexistence of Nb, S, Se, N and C elements (Fig. S10). Figure S11 shows the high-resolution Nb *3d* spectrum of NbSSe/NC and NbS_2_/NC samples, in which one can observe two peaks at ~ 207.0 and ~ 204.5 eV relating to the chemical environment of *3d*_*3/2*_* and 3d*_*5/2*_ of Nb^4+^, respectively, accompanied with a pair of shoulder peaks at ~ 211.1 and ~ 208.3 eV referring to Nb^5+^ [22, 23]. As shown in the S *2p* and Se *3p* high-resolution XPS spectrum (Fig. 2h), the spectra can be deconvoluted into four peaks, where a pair of peaks at 162.8 eV and 164.5 eV are attributed to *2p*_*3/2*_ and *2p*_*1/2*_ of S^2−^, and others at 161.7 and 165.8 eV belong to Se *2p*_*3/2*_ and Se *2p*_*1/3*_, respectively [24, 25]. Compared with that of NbS_2_/NC sample, the characteristic peaks of both Nb *3d* and S *2p* for the NbSSe/NC show a negative migration (~ 0.9 eV) toward lower binding energies, which are assigned by the less electronegative chemical environment due to the incorporation of Se^2−^ into NbS_2_ crystal lattice, thus decreasing energy binding of Nb–S. For the Se *3d* spectra of NbSSe/NC (Fig. S12), two typical peaks are centered at 55.5 and 54.8 eV, corresponding to valence feature of Se^2−^ (Nb–Se) [26]; the peak at 56.3 eV could be assigned to the formation of Se–C bonds [27]. Moreover, the existence of Se–C bonds also could be detected from the C *1 s* spectrum (Fig. S13), which indicate interface coupling between NbSSe nanosheets and carbon layer, ensuring the reinforced electrical conductivity and the structural stability of composites. The high-resolution N *1 s* spectrum suggests the doping of N atoms into the carbon layer with fitting peaks at 402.6 eV (graphitic-N), 400.1 eV (pyrrolic-N) and 399.4 eV (pyridinic-N), as shown in Fig. S14 [28]. Furthermore, the electric conductivities of NbSSe/NC and NbS_2_/NC composites were measured via a four-point probe method (Fig. S15). The NbSSe/NC exhibited a significantly enhanced electronic conductivity of 3.23 × 10^3^ S m^−1^ relative to that (1.12 × 10^3^ S m^−1^) of NbS_2_/NC, verifying the introduction of Se atoms endows NbS_2_ with high electrical conductivity [29]. The carbon content in NbSSe/NC was measured to be ~ 9.82% according to the thermogravimetric analysis (TGA, Fig. S16). It should be pointed out that the NbSSe/NC demonstrates a smaller specific surface area (100 m^2^ g^−1^) compared with NbS_2_/NC (125 m^2^ g^−1^), which should be attributed to the doping of heavy Se atoms (Fig. S17).

### Electrochemical Performance of NbSSe/NC Anode

**Fig. 3:**
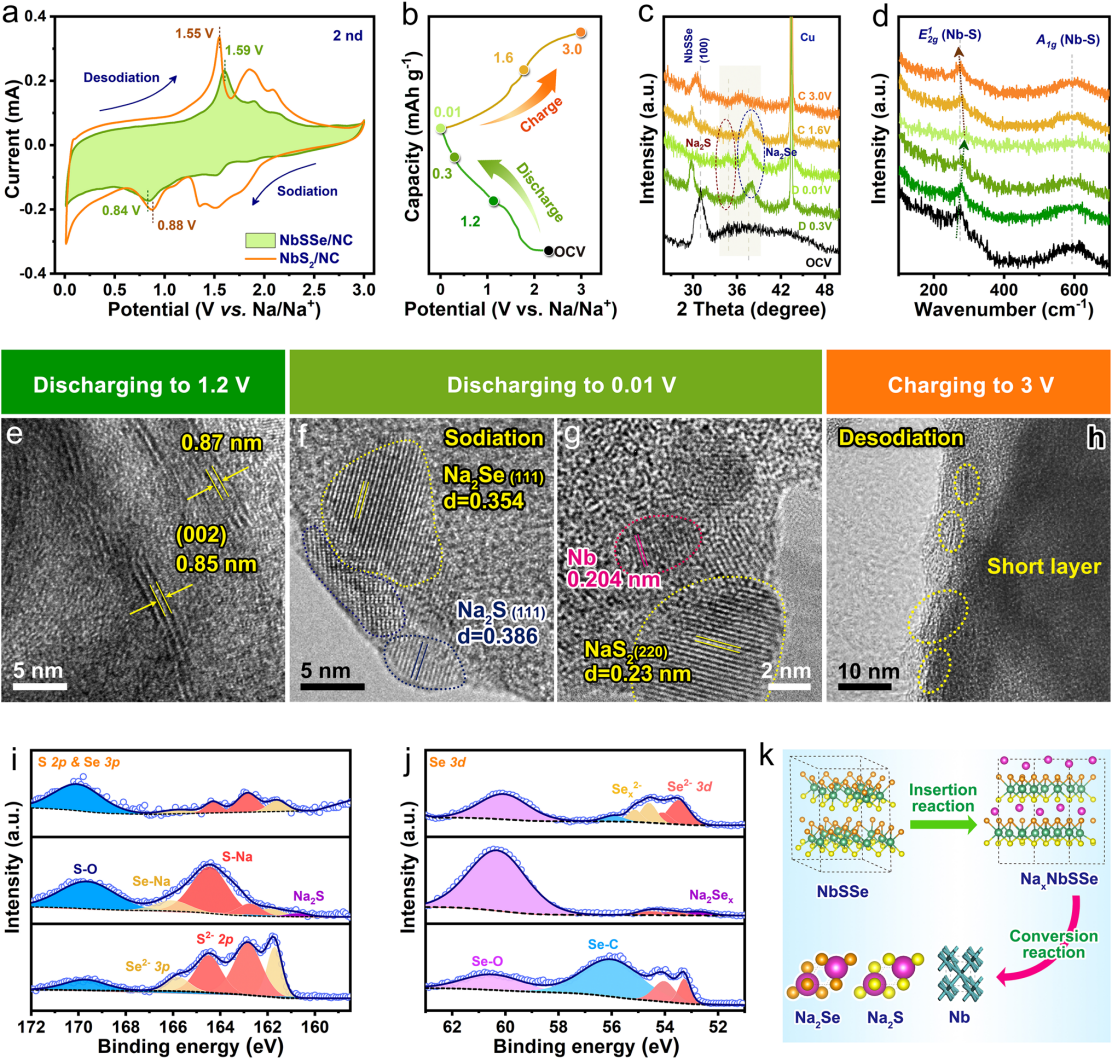
**a** CV curves during second cycle. **b** Initial charge-discharge curves. **c** Ex situ XRD. **d** Raman pattern coupled with corresponding charge/discharge curves. **e–h** Ex situ HRTEM images at various potentials during initial cycle. **i, j** Ex situ XPS pattern. **k** Schematic diagram of electrochemical mechanism for NbSSe/NC

To study the electrochemical properties of the NbSSe/NC nanosheets architecture for Na^+^ storage, the cyclic voltammogram (CV) and ex situ characterizations were conducted. As illustrated in the CV curves (Fig. 3a), a sharp strong peak at 1.51 V during the initial cycle is attributed to the Na^+^ intercalation into NbSSe/NC interlayers. A board peak at around 0.88 V is attributed to the conversion reaction with the formation of metallic Nb and Na_2_S and Na_2_Se. At the initial reverse scanning, three anodic peaks at about 1.55, 2.05 and 2.14 V are observed, which correspond to the processes of the reverse conversion reactions and the Na^+^ extraction process. Interestingly, the higher peak current density and smaller polarization voltage between conversion reaction can be observed for the NbSSe/NC electrode, further proving the introduction of Se can favorably promote the electrochemical reactivity (Fig. S18) [30]. The subsequent CV curves of NbSSe/NC are almost overlapped, implying a good stability of NbSSe/NC anode after the initial cycle (Fig. S19).

Furthermore, the structural transition and phase evolution of NbSSe/NC during the initial cycle were examined by ex situ XRD, Raman, XPS and HRTEM characterizations. Figure 3b-c shows the ex situ XRD patterns of NbSSe/NC for the initial cycle. Before discharging, the peak at 31.1° could be assigned to (100) crystal planes of NbSSe. When the cell discharges to 0.3 V, this peak negatively shifts to 29.8°, implying the lattice expansion due to the intercalation of Na^+^ into NbSSe forming intermediate product of Na_x_NbSSe; the new diffraction peaks at 34.8° and 37.5° synchronously emerge, which could be indexed to the formation of Na_2_S and Na_2_Se interphases, implying the occurrence of the conversion reaction [31, 32]. As the potential decreases to 0.01 V, the characteristic peaks belonging to NbSSe completely disappear. After recharging back to 3.0 V, the (100) peak reappears and shifts back to the pristine position, which suggests this process is reversible. According to the ex situ Raman spectroscopy (Fig. 3d), one can observe that the *E*_*2g*_ (Nb–S) vibration peak of NbSSe tends to decay and shift positively to a higher wavenumber during Na^+^ insertion and finally vanishes owing to the consecutive intercalation-conversion phase transition [33]. This peak gradually recovers again during the subsequent charging process, which is in accord with XRD results [34].

The phase evolution upon charging–discharging process was further confirmed by ex situ HRTEM (Fig. 3e-h). The (002) lattice plane space is expanded from ~ 0.65 to ~ 0.85 nm when the Na^+^ insertion (Fig. 3e), and materials would subsequently convert into metallic Nb, Na_2_S and Na_2_Se after discharging to 0.01 V (Fig. 3f-g) [15, 35]. When reversibly charged to 3.0 V, the interlayer structural nanosheets would re-emerge (Fig. 3h). The corresponding mechanisms were also investigated by ex situ XPS. As shown in the S region (Fig. 3i), for the fresh electrode, the doublet characteristic peaks at 161.7 and 162.9 eV represent the *2p*_*3/2*_ and *2p*_*1/2*_ orbitals of S^2−^ of metal sulfide. After discharging to 0.01 V, a new peak at a lower binding energy of 160.5 eV emerges, which indicates the formation of Na_2_S, and a slight peak of S^2−^ is detected, which might be assigned to the existence of Na_x_NbSSe [36]. With charging to 3.0 V, the peak of Na_2_S disappears and the intensity of S^2−^ peaks recovers again. The similar trend can also be observed in the binding energy of Se *2p* in the energy shifts and intensities (Fig. 3j), confirming a reversible Na^+^ storage capability. The above results reveal that the NbSSe/NC tends to follow an intercalation-conversion reaction during the cycling process, as schematically shown in Fig. 3k.

**Fig. 4:**
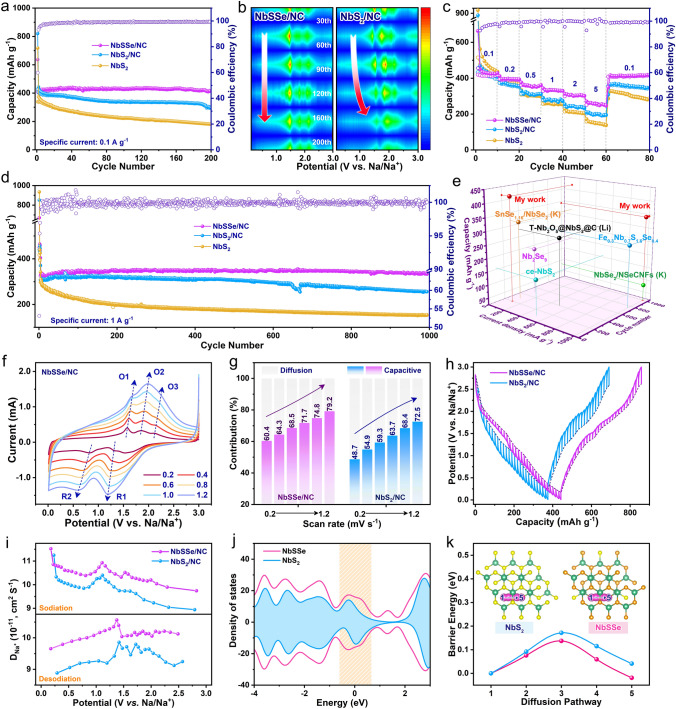
**a** Cycling performance at 0.1 A g^−1^ and **b** the corresponding dQ/dV curves from the 10th to 200th cycle for NbSSe/NC and NbS_2_/NC electrode. **c** Rate capability and **d** long-term cycling performance at 1 A g^−1^. **e** Survey of long-cycling stability of selective Nb-based material electrodes. **f** CV curves at various scan rates from 0.2 to 1.2 mV s^−1^, **g** normalized contribution ratio of capacitive and diffusion at different scan rates, **h** GITT curves and **i** corresponding Na^+^ diffusion coefficients of NbSSe/NC and NbS_2_/NC at 0.1 A g^−1^ after the 10th cycles. **j** Total density of states (DOS). **k** Schematic illustration for the diffusion path of Na^+^ in the NbS_2_ and NbSSe, and corresponding diffusion energy barrier curves

The electrochemical performance was measured by 2032-type half-cells with the metallic sodium as counter and reference electrode. Figure 4a presents cycling performance of Nb-based electrode at a current density of 0.1 A g^−1^, where NbSSe/NC electrode can deliver the first discharging and charging capacities of 645.1 and 419.8 mAh g^−1^ with the initial Coulombic efficiency (ICE) of 65.2%, which is much higher than that of NbS_2_/NC (51.9%), and NbS_2_ nanosheets (47.1%). The increased ICE of NbSSe/NC electrode further demonstrates the improved reversibility, majorly because the bonding energy of Nb–Se bond is weaker than that of Nb–S bond. Accordingly, the NbSSe/NC electrode shows durable cycling stability, where the specific capacity can maintain around 413.5 mAh g^−1^ after 200 cycles with a capacity retention of 96.4% relative to the second-cycle capacity, which outperforms those of the NbS_2_/NC (293.6 mAh g^−1^, 67.6%) and NbS_2_ nanosheets (181 mAh g^−1^,48.3%). Figures 4b and S20 display the dQ/dV plots of both Nb-based electrodes, where the redox peaks of NbSSe/NC can well maintain even after 200 cycles. In contrast, there is significant variation in the redox peaks for NbS_2_/NC electrode, which are possibly attributed to the loss of active materials due to the shuttle effect of polysulfides [28].

The rate capability at various current densities is further studied, as shown in Fig. 4c. The NbSSe/NC electrode can deliver the specific capacities of 425.3, 396.2, 356.6, 333.6 and 307.1 mAh g^−1^ at current densities from 0.1 to 2.0 A g^−1^, respectively, which display a slightly attenuated tendency with the increase in specific current. Notably, even at the high rate of 5.0 A g^−1^, this value can still maintain as high as 262.4 mAh g^−1^, and the specific capacity can recover to 414.6 mAh g^−1^ when the current density returns to 0.1 A g^−1^. In contrast, under the same testing conditions, both NbS_2_/NC and NbS_2_ electrodes exhibit a rapid capacity decay from 452.5 to 201.6 mAh g^−1^ and 478.8 to 148.5 mAh g^−1^, respectively. Additionally, the stable GCD profiles and slowly increased electrochemical polarization phenomenon of NbSSe/NC with the increasing specific current further confirm the excellent Na^+^ capture ability (Fig. S21). To the best of our knowledge, the NbSSe/NC shows improved rate capability relative to the majority of recently reported works for NbS_2_-, NbSe_2_- and NbSSe-based anodes for Na^+^ storage (Fig. S22). The long-term cycling performance was tested at 1.0 A g^−1^, as shown in Fig. 4d. The reversible capacity of NbSSe/NC can maintain over 347.8 mAh g^−1^ with a capacity retention of 95.6% after 1,000 cycles, being indicative of robust stability (Fig. S23), and is distinctly better than those of the NbS_2_/NC (76.1%) and NbS_2_ (49.2%). In addition, the NbSSe/NC also delivers excellent cycling stability after rate performance measurements, further implying the highly reversible Na^+^ storage behavior (Fig. S24). The excellent cycling performance of NbSSe/NC shows competitive to other reported Nb-based anodes for SIBs (Fig. 4e) [8, 21, 23, 37–40].

### Kinetic Analysis of NbSSe/NC Anode

In order to in-depth shed light on the enhanced Na^+^ storage kinetics of the NbSSe/NC anode, the CV measurement at different scan rates was performed (Fig. 4f). As shown in Fig. 4g, the capacitive contribution percentage is gradually increased with enhancing of scan rates, and one can observe that the capacitive-controlled contributions in NbSSe/NC are higher than that of NbS_2_/NC at the same scan rate (Figs. S25–S26), demonstrating the improved reaction kinetics in NbSSe/NC anode [41]. The galvanostatic intermittent titration technique (GITT) test was performed to in situ evaluate interfacial reaction resistances of Nb-based electrodes during the sodiation and desodiation process (Figs. 4h-i and S27) [42]. It can be seen that the NbSSe/NC presents a slight voltage hysteresis corresponding to a weak electrochemical polarization. Moreover, the NbSSe/NC shows much higher Na^+^ diffusion coefficients (D_Na_) than NbS_2_/NC, uncovering the faster Na^+^ diffusion property thanks to the enlarged interlayer space in NbSSe/NC composite. The fast kinetics can be attributed to the improved conductivity, as revealed by electrochemical impedance spectroscopy (EIS) analysis (Fig. S28). The charge transfer resistance (Rct) for NbSSe/NC is reduced value when compared with NbS_2_/NC [43]. Moreover, the calculated diffusion coefficient of NbSSe/NC (6.35 × 10^–10^ cm^2^ s^−1^) is higher than that of NbS_2_/NC (1.38 × 10^–12^ cm^2^ s^−1^), revealing a speedy Na^+^ diffusion in NbSSe/NC. Furthermore, the density of states (DOS) value of NbSSe is higher than that of NbS_2_, which indicates the introduction of anion defect can optimize the electronic structure and facilitate electron transfer efficiency, agreeing with the electrical conductivity result (Fig. 4j) [44]. As indicated in Figs. 4k and S29, the diffusion energy profiles simulate the energy barriers of Na^+^ migration in the interlayers of both models, where a lower value for NbSSe/NC suggests a positive effect of the enlarged interlayers and anion defects toward the optimization of Na^+^ volume-phase diffusion kinetics and thus achieving a favorable rate performance [45].

Based on the above results, the NbSSe/NC presents a set of merits as the anode material for Na^+^ storage: (i) the introduction of anion defects presents a cooperative effect on the charge self-regulation, increasing abundant active sites and enhancing intrinsic electrical conductivity; (ii) the formation of weak Nb–Se ionic bonds is prone to separate than Nb–S bands, which is favored to improve the reaction reversibility; (iii) the expended lattice spacing provides broads Na^+^ diffusion paths and lowers Na-ion diffusion energy barrier; and iv) besides, the N-doped carbon protect layer is beneficial to relieve the volume expansion and maintain the structural stability. Therefore, these structural and componential merits of NbSSe/NC endow it with fast charge-storage dynamics for achieving high-performance SDIBs.

### Electrochemical Performance Tests in SDIBs

**Fig. 5:**
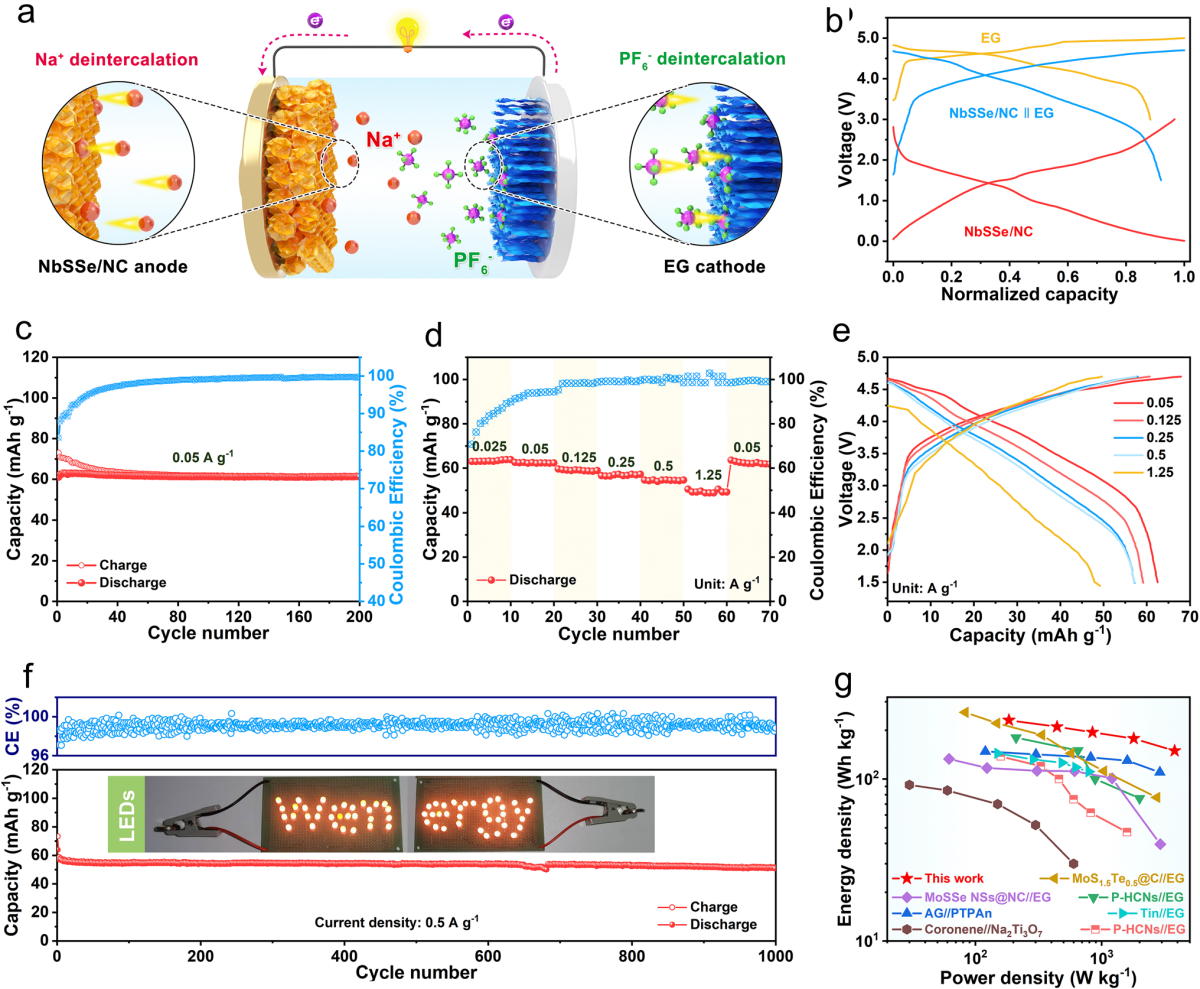
The electrochemical performance of SDIBs. **a** Schematic diagram of NbSSe/NC//EG full cell. **b** Charge–discharge profiles of NbSSe/NC anode, EG cathode and full cell. **c** Cycling performance and **d** rate capability of the full cells. **e** Galvanostatic charge–discharge voltage profiles of full cell at various rates. **f** Long cycling stability of full cells at 0.5 A g^−1^. Inset in **f**: The digital photographs of the “Wenergy” logo composing of 64 LEDs light up by two cells. **g** Comparison of the energy density of NbSSe/NC//EG full cell with the reported SDIBs

The Na dual-ion full cells were assembled by employing the pre-sodiated NbSSe/NC as anode and expanded graphite (EG) as the cathode, which are schematically presented in Fig. 5a. The structural characteristics and electrochemical performance of EG are shown in Figs. S30–S31 [46]. Figure 5b shows the GCD curves of voltage *vs.* normalized capacity for NbSSe/NC anode and EG cathode. According to the charge balance criterion between individual material levels, the voltage cutoff window is selected between 1.5 and 4.8 V, and the electrode mass ratio of anode/cathode is ~ 1:4 (Fig. S32) [47]. As depicted in Fig. 5c, the dual-ion full cell displays a high reversible capacity of 62 mAh g^−1^ with a capacity retention of 87.1% over 200 cycles at 0.05 A g^−1^. (The specific capacity is calculated based on the total mass of anode and cathode.) More importantly, the medium discharge voltage could be stably maintained at 3.70 V during the cycling process (Fig. S33), suggesting their high-work voltage feature. Figure 5d displays the rate performance at the various current density, and the delivered capacities of full cells are 62.5, 59.7, 57.2, 54.6 and 58.8 mAh g^−1^ at the current rates of 0.05, 0.125, 0.25, 0.5 and 1.25 A g^−1^, respectively. Moreover, the GCD curves at different current densities show low polarization phenomenon (Fig. 5e), verifying the excellent rate ability of full cell. The long-term cycling stability of full cell at a high current density of 1 A g^−1^ is illustrated in Fig. 5f. After a long-time cycling over 1,000 cycles, the reversible capacity of full cell can be well maintained at 51.7 mAh g^−1^, corresponding to a high capacity retention of 89.1%. In addition, this device can successfully light up a simple “Wenergy” logo pattern consisting of light-emitting diode (LED) bulbs, which embodies its potential practical value (inset in Fig. 5f). Figure 5g displays the Ragone plot of the Na-DIBs full cell based on the total mass of the anode and the cathode. As a result, the NbSSe/NC//EG full cells achieve a maximum specific energy density of 230.6 Wh kg^−1^ at a current density of 0.05 A g^−1^, which is comparable to and even exceeds those of some representative sodium-based dual-ions full batteries reported in the literature (Fig. S34) [3, 48–52]. Additionally, the cycling performance comparison between this work and previously reported SDIBs is displayed in Tables S2 and S3, which indicates its potential feasibility as a high-performance energy storage device for future application.

## Conclusions

In summary, we reported single-phase NbSSe nanosheets hybrids architecture consisting of N-doped carbon film-modified few-layer sheets as building units. The anionic substitution of Se atoms into the NbS_2_ lattice would not only broaden interlayer spacing and enhance intrinsic electronic conductivity, but also generate abundant structural defects, and provides rich active sites for Na^+^ ion storage, significantly promoting the electron/ions migration kinetic, electrochemical reaction reversibility, as well as pseudocapacitive storage effect. With these merits, the as-prepared NbSSe/NC anode can deliver a reversible capacity of as high as 414.6 mAh g^−1^ at 0.1 A g^−1^ over 100 cycles and long-term stable cyclability with 95.6% capacity retention after 1,000 cycles at 1 A g^−1^. The NbSSe/C was also verified to be practically potential in a full sodium-based dual-ion battery cell by exhibiting a high work voltage and stable capacity output over 1000 cycles, and high energy density of 230.6 Wh kg^−1^. Such defect engineering strategy in this work provides a new strategy for the construction of high-performance anode materials and highlights their applicability prospects in sodium-based energy storage devices.

### Supplementary Information

Below is the link to the electronic supplementary material.

Supplementary file1 (PDF 2755 kb)
