# Supplementary material for: Anion Defects Engineering of Ternary Nb-Based Chalcogenide Anodes Toward High-Performance Sodium-Based Dual-Ion Batteries
Source: Nanomicro Lett. 2023 Apr 15;15:104. doi: 10.1007/s40820-023-01070-0 (PMC10105816; doi:10.1007/s40820-023-01070-0)
Supplement: Supplementary file 1 — Supplementary file1 (PDF 2755 kb) [file 40820_2023_1070_MOESM1_ESM.pdf]

Supporting Information for

Anion Defects Engineering of Ternary Nb-Based Chalcogenide Anodes towards High-Performance Sodium-Based Dual-Ion Batteries

Yangjie Liu[1,2], Min Qiu[2,3], Xiang Hu[1,2], Jun Yuan[1,2], Weilu liao[2], Liangmei Sheng[4], Yuhua Chen[4], Yongmin Wu[4], Hongbing Zhan[1,*], Zhenhai Wen[2,*]

[1] College of Materials Science and Engineering, Fuzhou University, Fuzhou 350108, P. R. China

[2] CAS Key Laboratory of Design and Assembly of Functional Nanostructures, and Fujian Provincial Key Laboratory of Materials and Techniques toward Hydrogen Energy, Fujian Institute of Research on the Structure of Matter, Chinese Academy of Sciences, Fuzhou, Fujian 350002, P. R. China

[3] Fujian Normal University, Fuzhou 350108, P. R. China

[4] State Key Laboratory of Space Power-sources Technology, Shanghai Institute of Space Power Sources, 2965 Dongchuan Road, Shanghai 200245, P.R. China

* Corresponding authors. E-mail: hbzhan@fzu.edu.cn (Hongbing Zhan), wen@fjirsm.ac.cn (Zhenhai Wen)

S1 Calculation of b Value

The b value is applied to evaluate the pseudocapacitive behavior of electrode. According to the power-law relation between the sweep scan rate (v) and the peak current (i), Eqs. S1 and S2 can be provided:

$$i = av^b \qquad \text{(S1)}$$
$$\log(i) = b\log(v) + \log(a) \qquad \text{(S2)}$$

in which the b-value of 0.5 or 1.0 indicates a fully diffusion-dominated or surface-capacitive process, respectively.

S2 Calculation of Capacitive Contribution

Quantitatively, the capacitive-dominated contribution can be separated based on the current response (i) at a fixed voltage (v), according to the Eqs. S3 and S4:

$$i(\mathrm{V}) = k_1 v + k_2 v^{1/2} \qquad \text{(S3)}$$
$$i(\mathrm{V})/v^{1/2} = k_1 v^{1/2} + k_2 \qquad \text{(S4)}$$

where k_1 and k_2 are adjustable parameters, the k_1v stands for capacitive-controlled process, and the $k_2v^{1/2}$ represents ionic-diffusion controlled process.

S3 Calculation of Na^+ Diffusion Coefficient (D_{Na+})

Galvanostatic intermittent titration technique (GITT) measurement during the 10th cycle is utilized to reveal the Na^+ diffusion coefficient (D_{Na+}) in the WS_2/C@CNTs cathode. By virtue of the linear relationship of the voltage variation (ΔE_τ) and $\tau^{1/2}$ (Fig. S27), the D_{Na+} can be determined based on the following Eq. S5:

$$D_{Na^+}=\frac{4}{\pi\tau}\left(\frac{n_m V_m}{S}\right)^2\left(\frac{\Delta E_s}{\Delta E_\tau}\right)^2 \quad \text{(S5)}$$

where τ is the duration of the current pulse; nm and V_m are the mole number (mol) and molar volume (cm^3 mol^{-1}); S is the total contacting area between electrode and electrolyte; ΔE_s is the voltage change between two adjacent equilibrium states; and ΔE_τ is the voltage change induced by the galvanostatic charge/discharge.

S4 Calculation of the Specific Energy and Power (based on the total mass of both anode and cathode materials):

The cell-level specific energy E and specific power P are calculated according to the following Eqs. S6, S7:

$$E=\int_{t_1}^{t_2} VI\,dt=\frac{V_{max}+V_{min}}{2}\times It\times\frac{1}{3600} \quad \text{(S6)}$$

$$P=\frac{3600\times E}{t} \quad \text{(S7)}$$

where t (s) is the discharge time, I (A g^{-1}) is charge/discharge current, V_{max} (V) is the discharge potential excluding the IR drop and V_{min} (V) is the potential at the end of discharge voltages, E is the specific energy (Wh kg^{-1}) and P is the specific power (W kg^{-1}).

Table S1 Contents of Nb, Se, S, and C in NbSSe/NC and NbS_2/NC

Element	NbSSe/NC (wt%)	NbS_2/NC (wt%)
Nb[a)]	42.5	51.2
Se[a)]	32.7	/
S[b)]	13.5	34.5
C[b)]	11.3	14.3

a) The element results were analyzed by ICP-AES.
b) The element results were analyzed by EA.

S5 Supplementary Figures and Tables

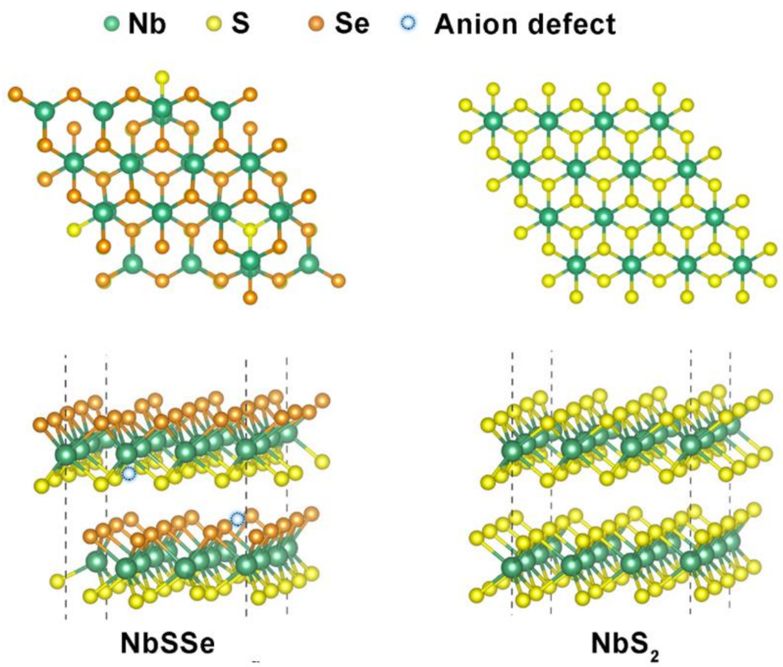

Fig. S1 Top and side view of the optimized structure of (**a**) NbSSe and (**b**) NbS_2

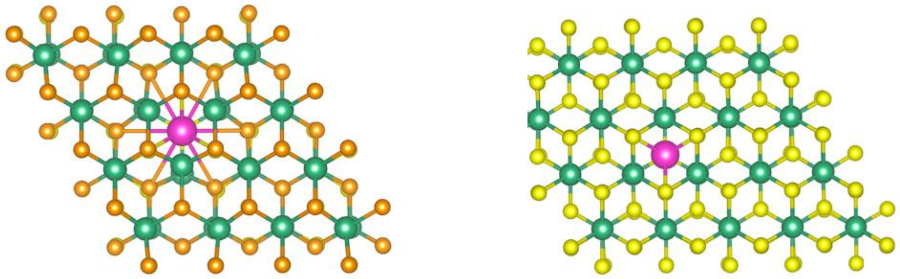

Fig. S2 Top illustration of simulations for one adsorbed Na^+ in the (left) NbSSe and (right) NbS_2

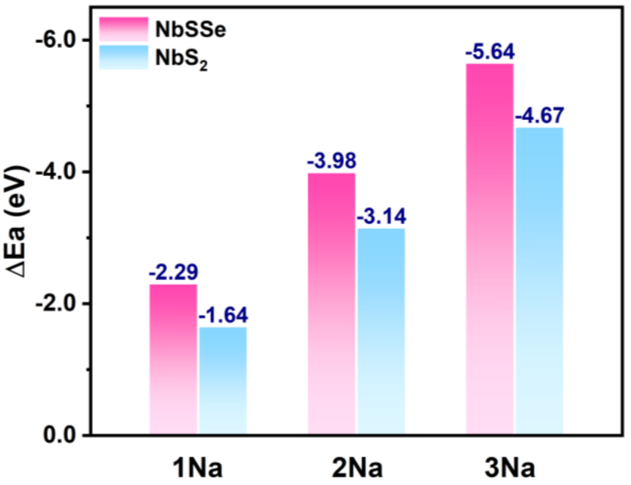

Fig. S3 The adsorption energy (ΔEa) for Na^+ ions in the NbSSe and NbS_2

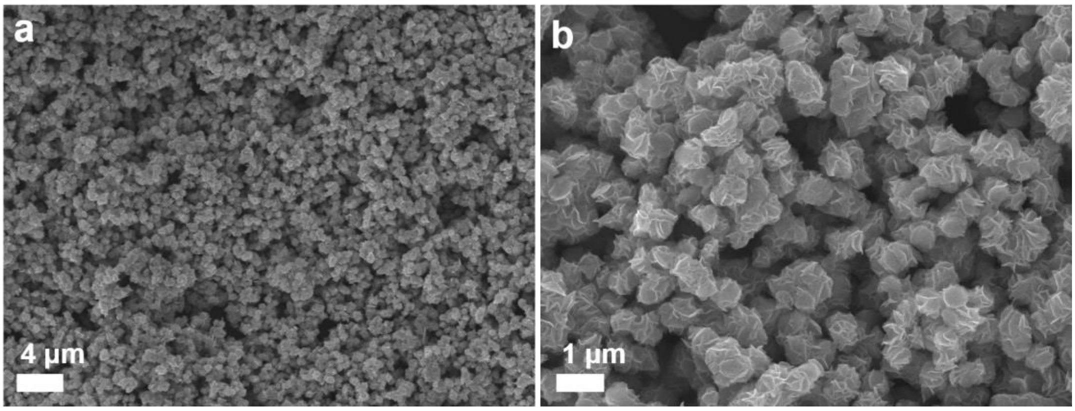

Fig. S4 The SEM images of NbS_2-OA

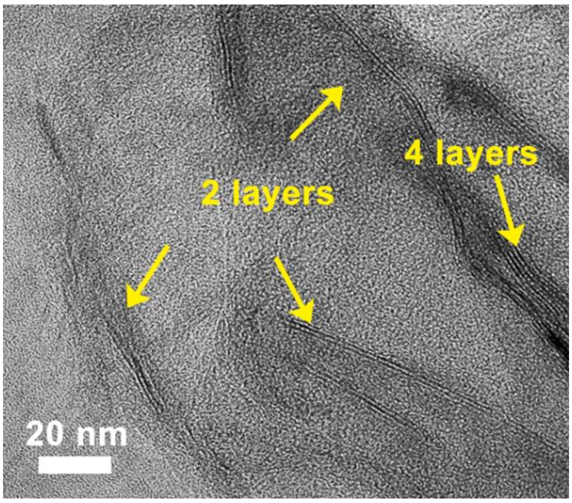

Fig. S5 The HRTEM images of NbSSe/NC

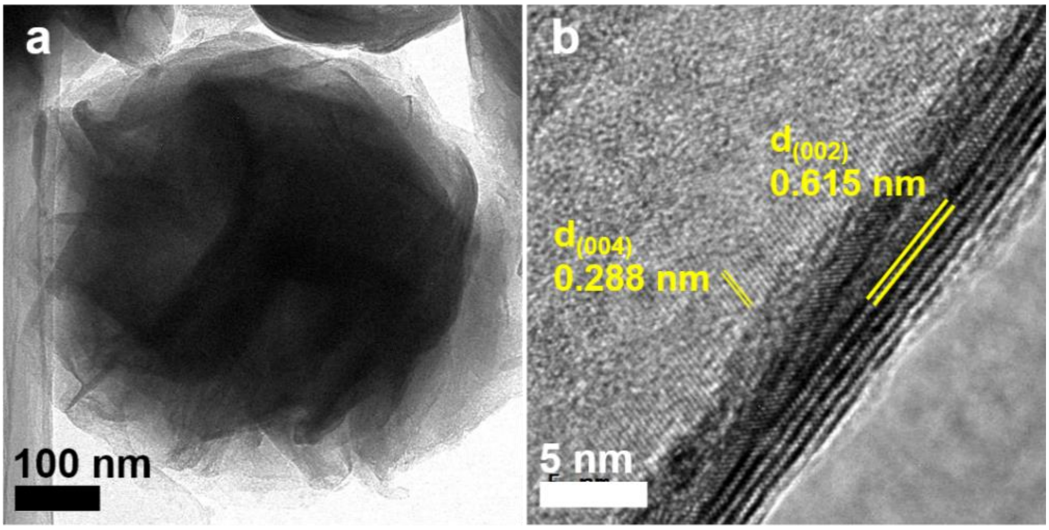

Fig. S6 The SEM images of NbS_2/NC

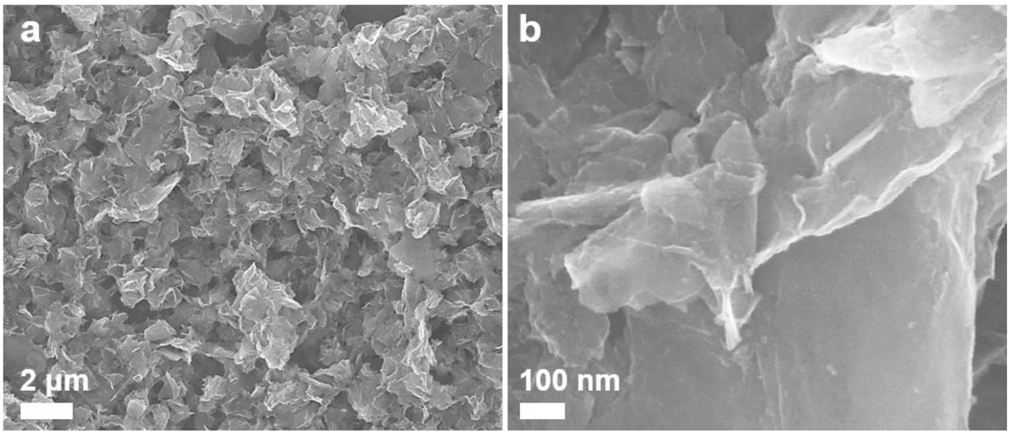

Fig. S7 The SEM images of NbS_2

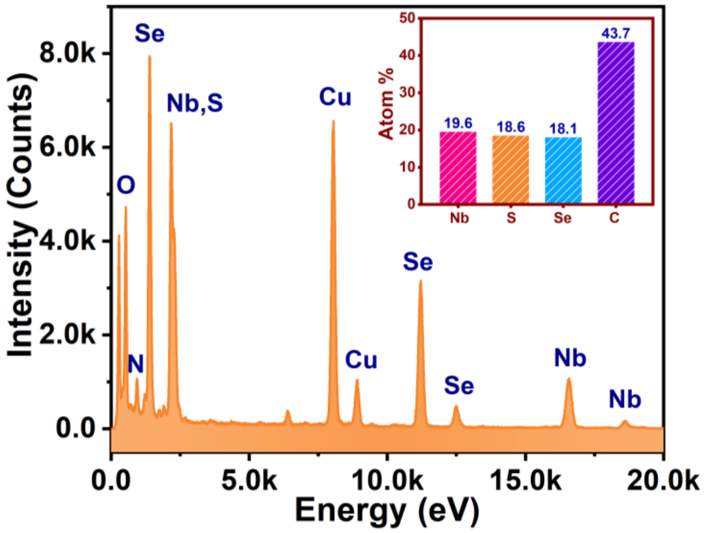

Fig. S8 The EDS data of NbSSe/NC

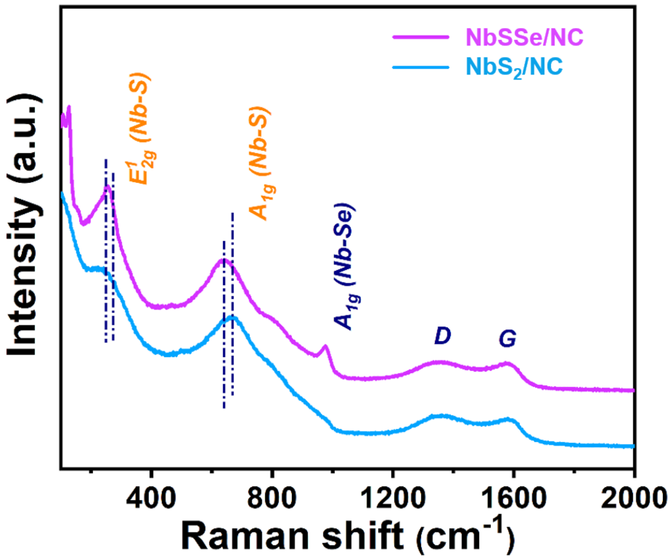

Fig. S9 The Raman spectroscopy of NbSSe/NC and NbS_2/NC

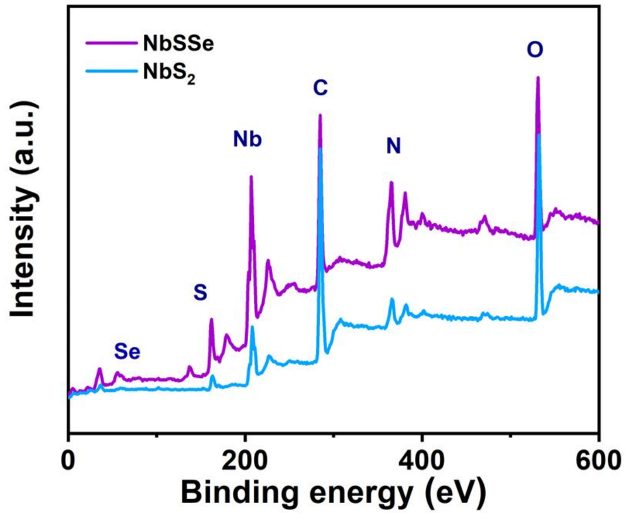

Fig. S10 The survey XPS spectrum of NbSSe/NC and NbS_2/NC

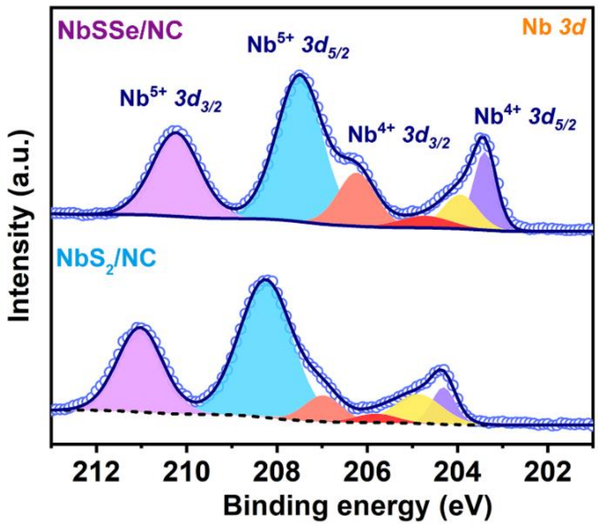

Fig. S11 The Nb *3d* high-resolution XPS spectrum of NbSSe/NC

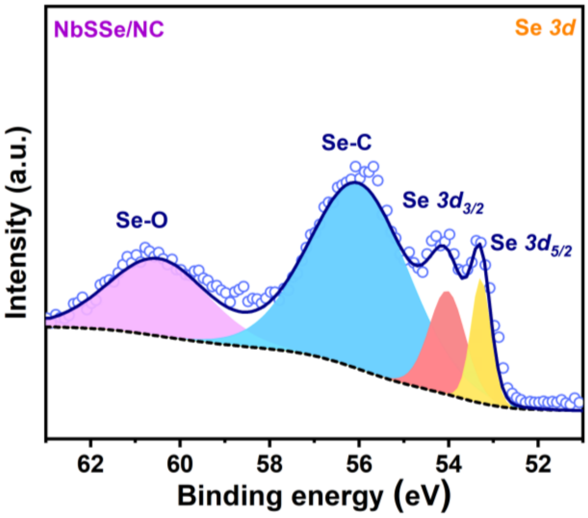

Fig. S12 The Se *3d* high-resolution XPS spectrum of NbSSe/NC

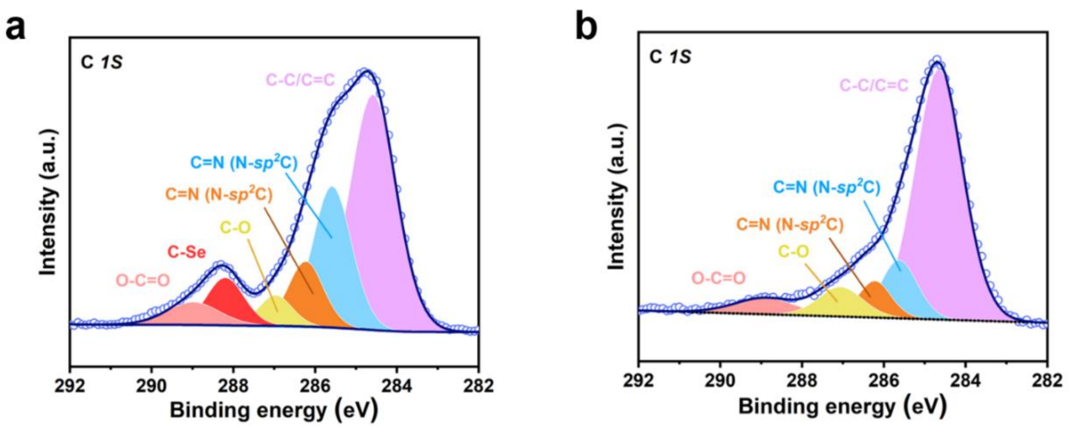

Fig. S13 The C *1s* XPS spectrum of NbSSe/NC (**a**) and NbS_2@NC (**b**)

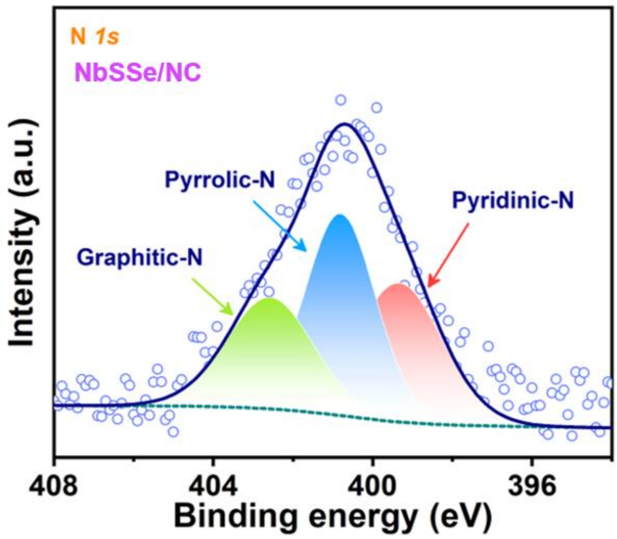

Fig. S14 The N *1s* XPS spectrum of NbSSe/NC

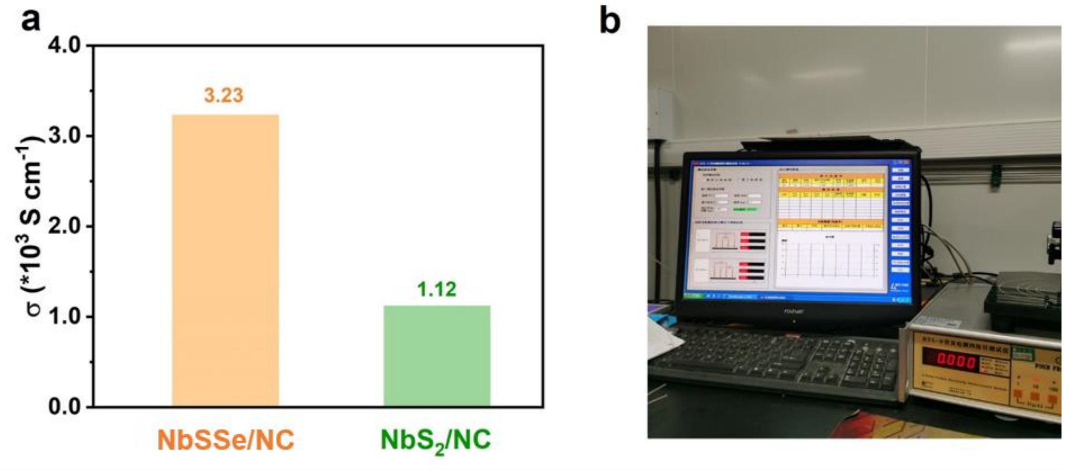

Fig. S15 (**a**) Electrical conductivity of the two materials (NbSSe/NC and NbS_2/NC) and (**b**) digital photography of four-point probe testing instrument

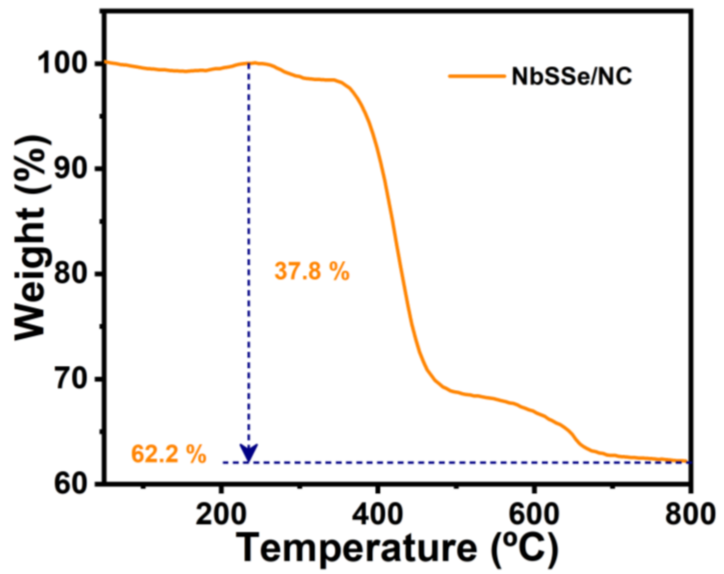

Fig. S16 Thermogravimetric analysis curves of NbSSe/NC

Based on the transformation of NbSSe/NC after the TGA test as shown below,

$$10NbS_{0.9}Se_{0.9} + 30.5O_2 = 5Nb_2O_5 + 9SO_2\uparrow + 9SeO_2\uparrow$$

The carbon content in NbSSe/NC is calculated by equation:

$$c=9.82\%$$

m represents the total mass of NbSSe/NC, c is the percentage composition of carbon in the NbSSe/NC.

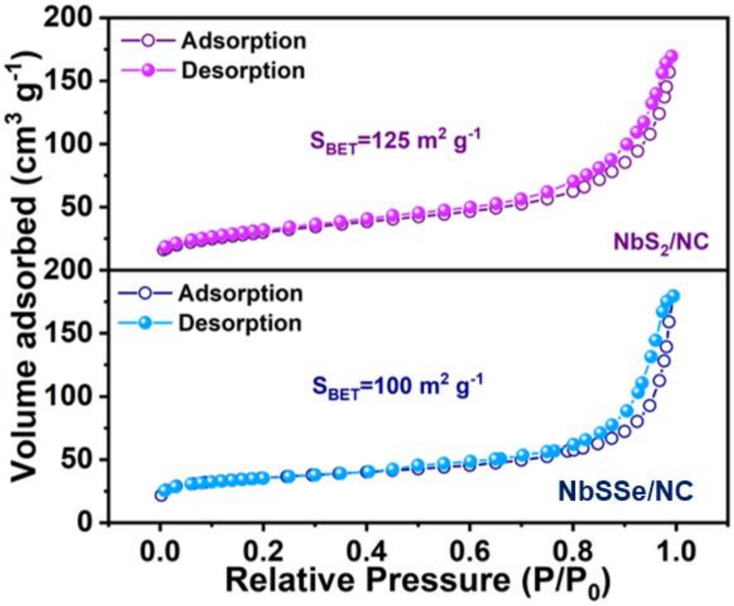

Fig. S17 Nitrogen adsorption-desorption isothermal curves for NbSSe/NC (**a**) and NbS_2@NC (**b**)

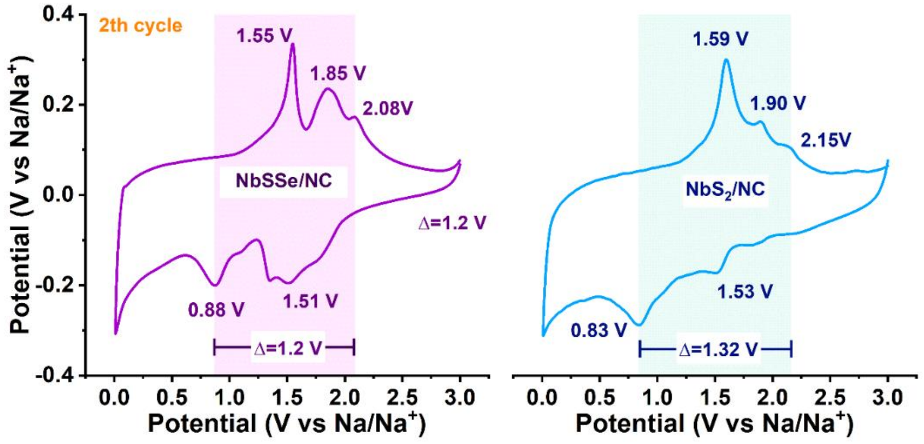

Fig. S18 The CV curves of 2nd cycle for NbSSe/NC (**a**) and NbS_2@NC (**b**)

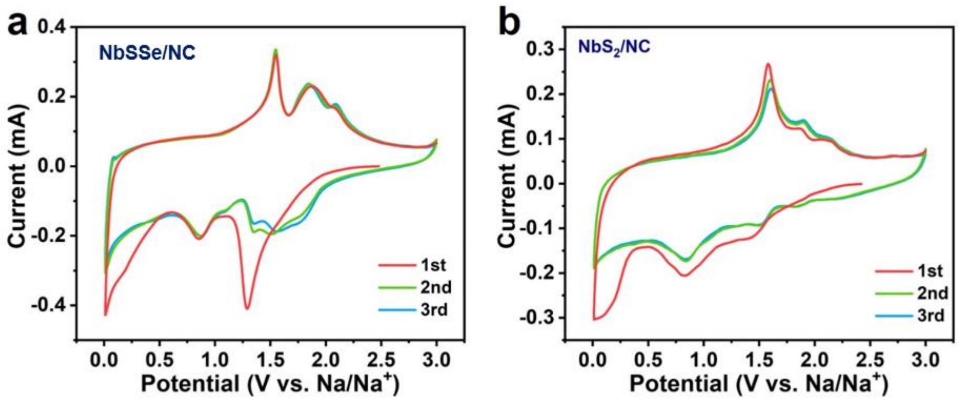

Fig. S19 The CV curves of initial 3 cycles for NbSSe/NC (**a**) and NbS_2@NC (**b**)

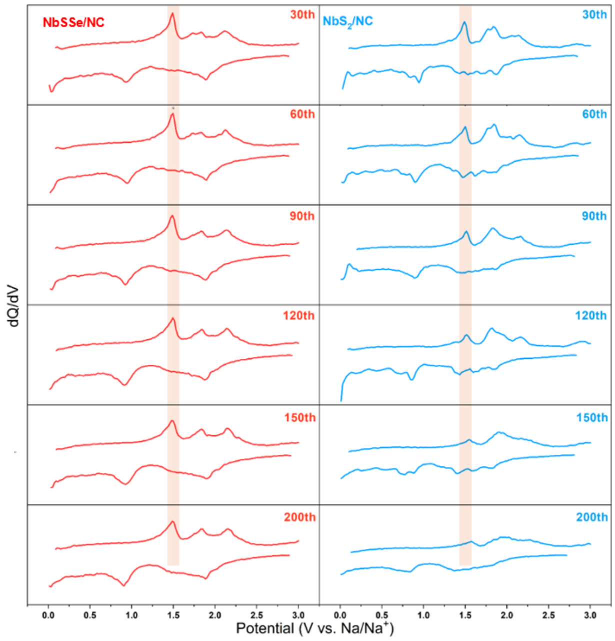

Fig. S20 The dQ/dV plots of NbSSe/NC and NbS_2/NC

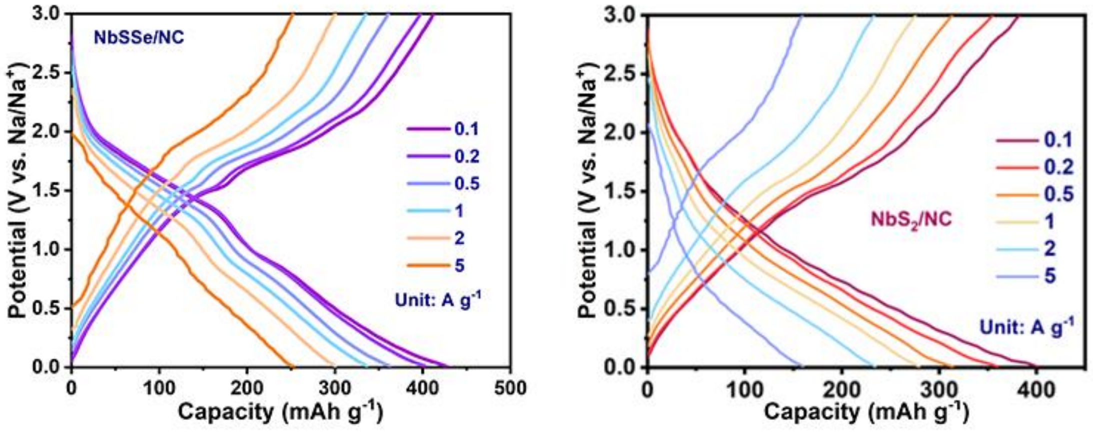

Fig. S21 The GCD profiles of different rates for NbSSe/NC and NbS_2/NC

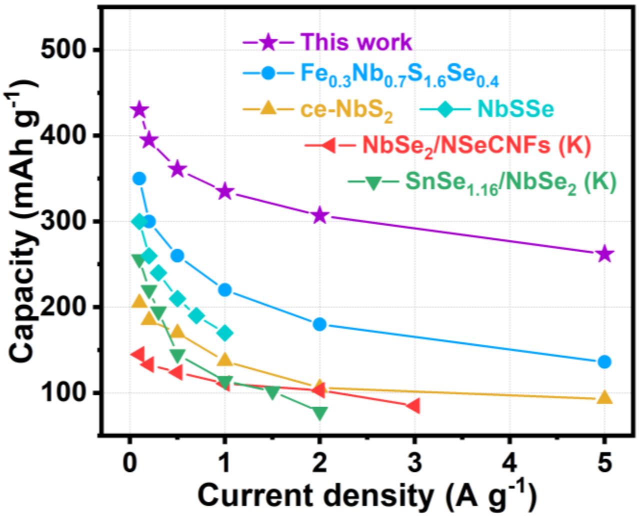

Fig. S22 The comparison of rate performance between the NbSSe/NC and reported Nb-based anode

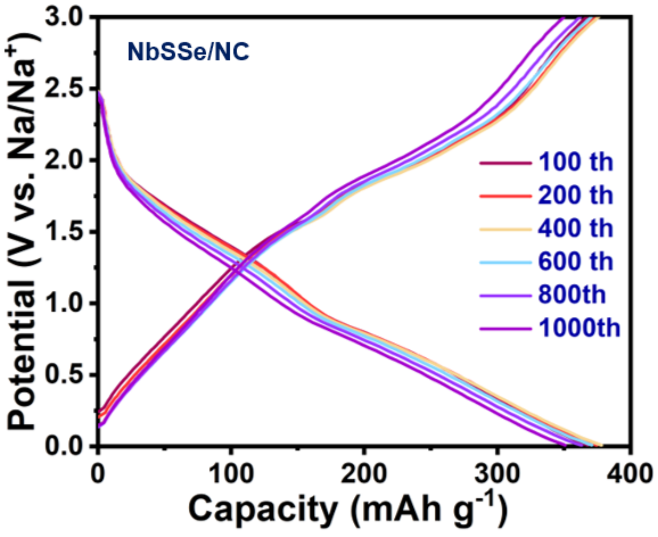

Fig. S23 The GCD profiles at different cycles for NbSSe/NC at 1 A g^{-1}

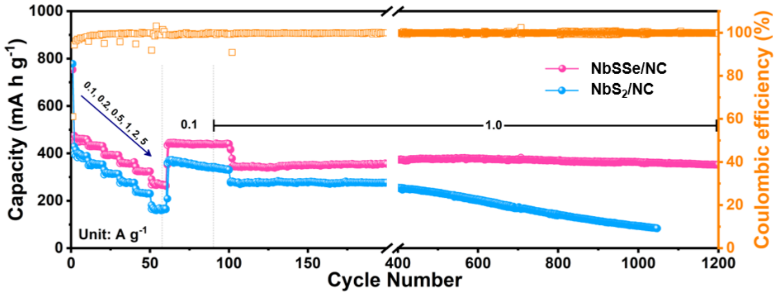

Fig. S24 The rate performance of NbSSe/NC and NbS_2/NC electrodes

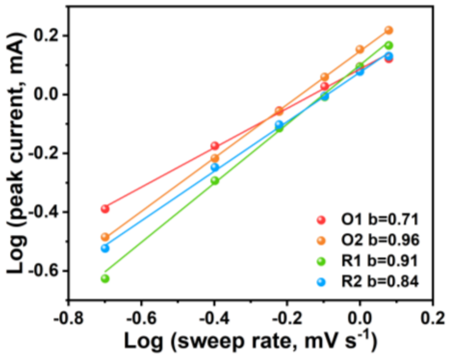

Fig. S25 Log (i) versus log (v) plots at different redox peaks of the NbSSe/NC

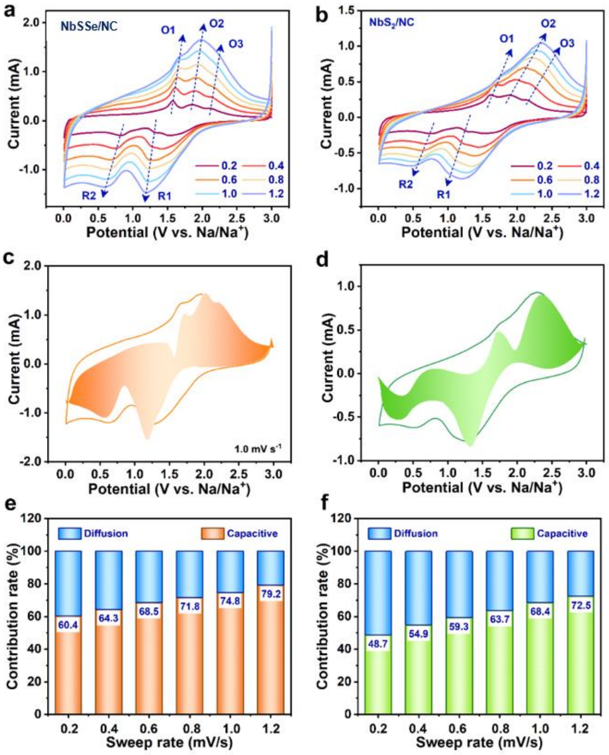

Fig. S26 (**a, b**) CV curves at various scan rates from 0.2 to 1.2 mV s^{-1}, (**c, d**) Capacitive contribution at 1.0 mV s^{-1}, (**e, f**) the percentages of capacitive and diffusion-controlled capacities at different scan rates of the NbSSe/NC (**a, c, e**) and NbS_2@NC (**b, d, f**)

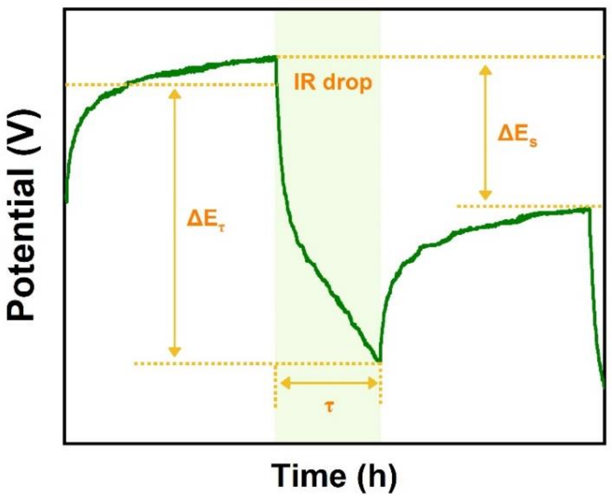

Fig. S27 Several key parameters for ion diffusion coefficient calculation according to GITT curves

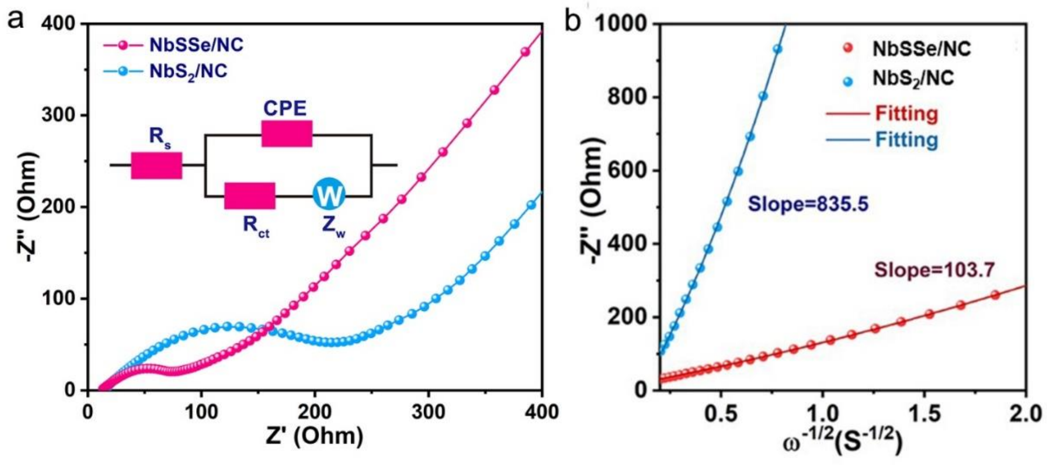

Fig. S28 Electrochemical impedance spectra of both Nb-based samples after 50 cycles

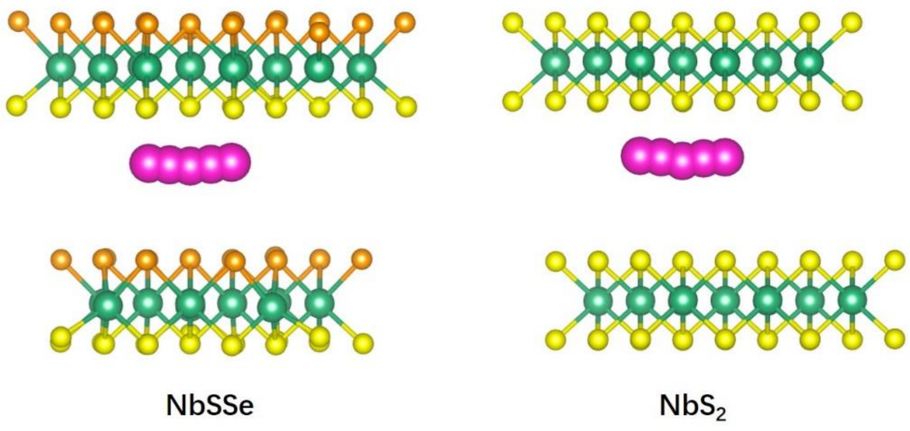

Fig. S29 Side illustration of simulations for the diffusion path of Na^+ in the NbSSe and NbS_2

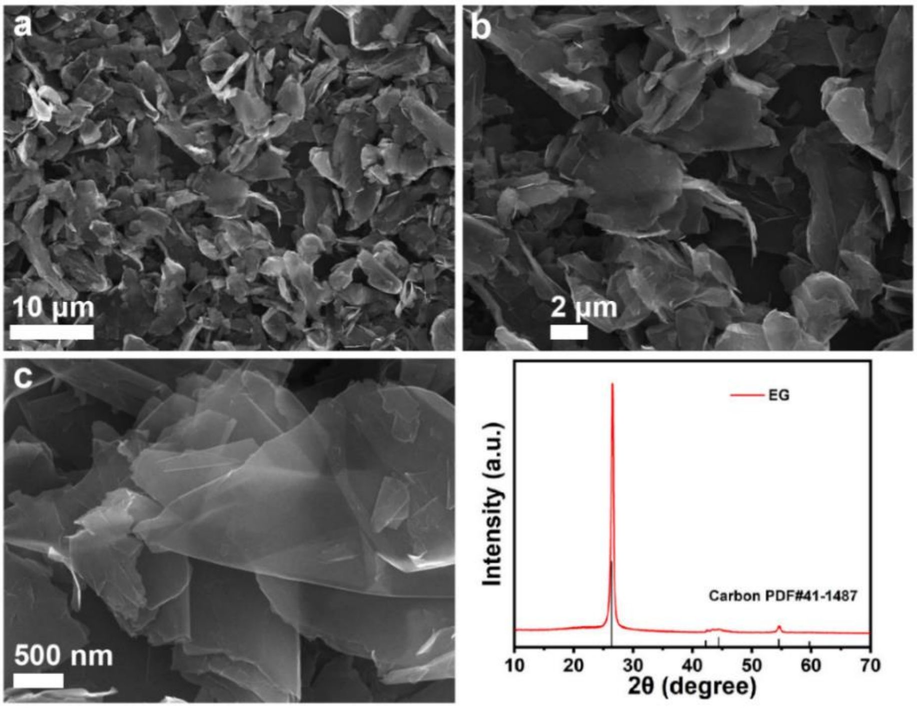

Fig. S30 The SEM images and XRD data of EG cathode

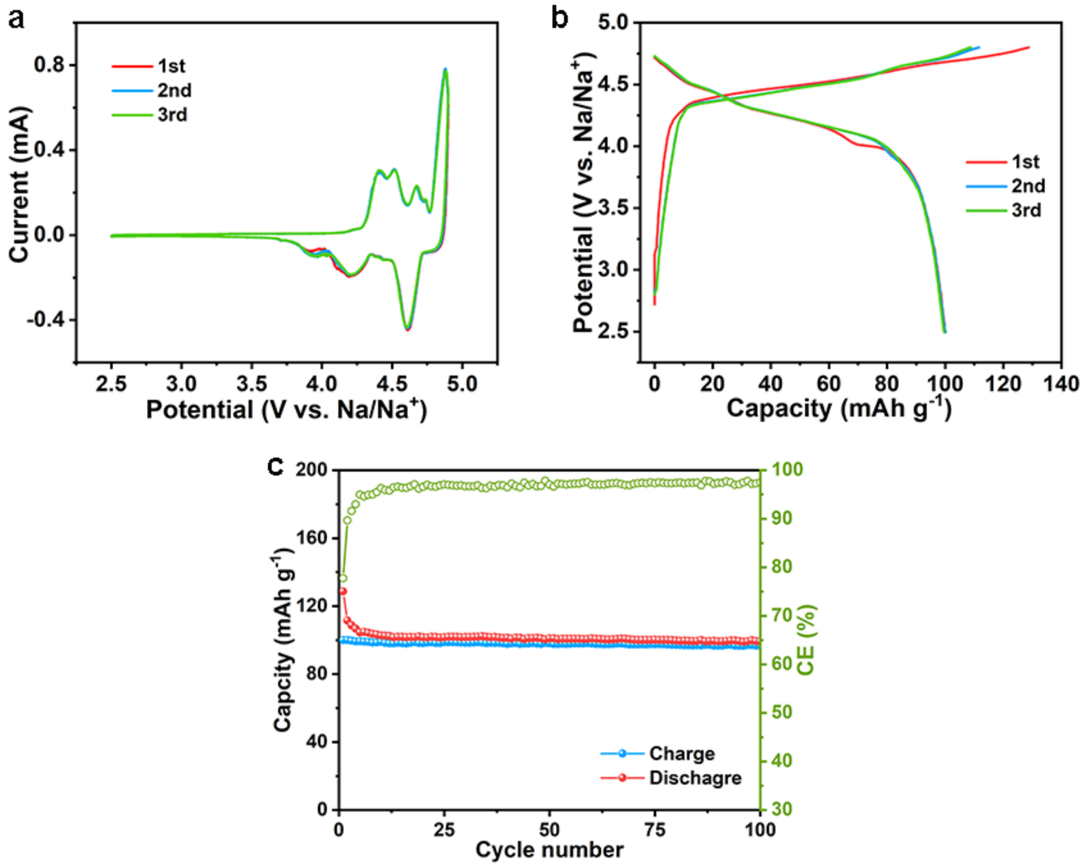

Fig. S31 The electrochemical performance of EG//Na half-cell

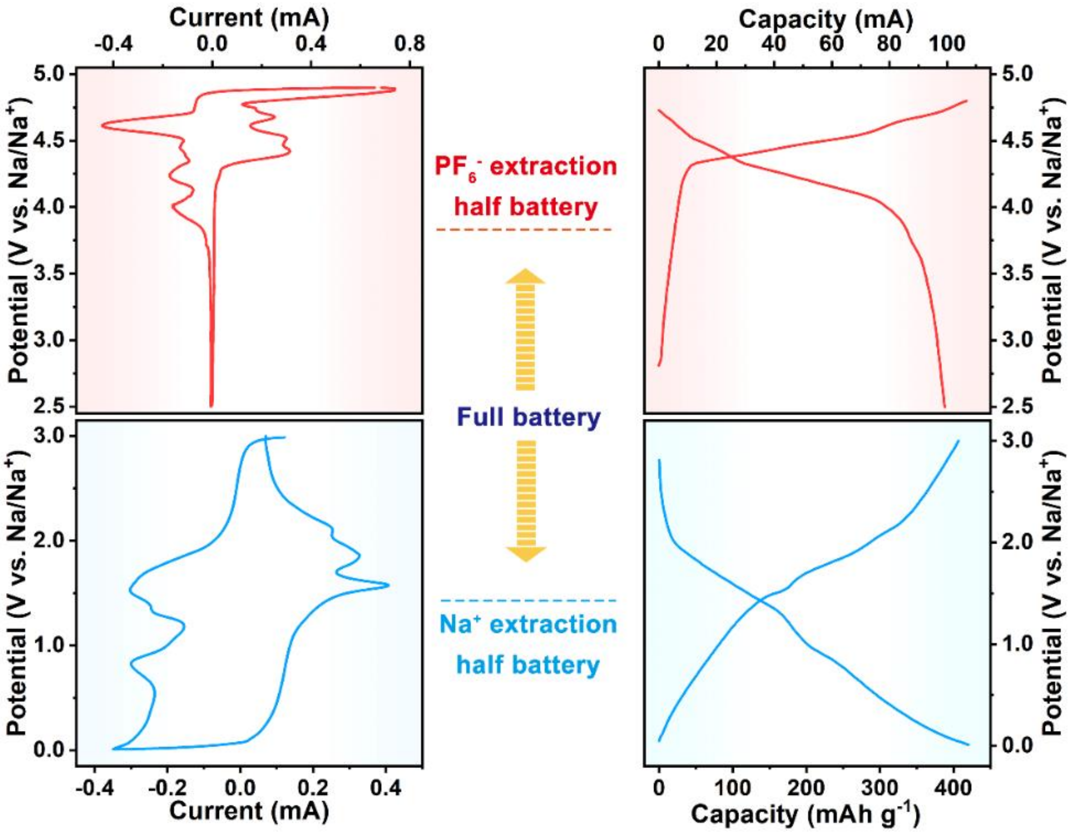

Fig. S32 CV and charge/discharge profiles (b,c) of the NbSSe/NC and EG half-cells

In order to obtain the optimized electrochemical performance, it was a key factor to balance the capacity between cathode and anode. The NbSSe/NC anode showed a specific capacity of around 420 mAh g^{-1}, and the EG cathode exhibited a specific capacity of around 100 mAh g^{-1}, thus the mass ratio of the EG cathode to NbSSe/NC anode was optimized to be 4:1 according to the charge balance Eq. S8:

$$C_{cathode} \times m_{cathode} = C_{anode} \times m_{anode} \quad \text{(S8)}$$

where C (mAh g^{-1}) and m (mg) are the specific capacity of both electrodes and the mass of active materials, respectively. Therefore, the mass ratio between cathode and anode was calculated to be 4:1.

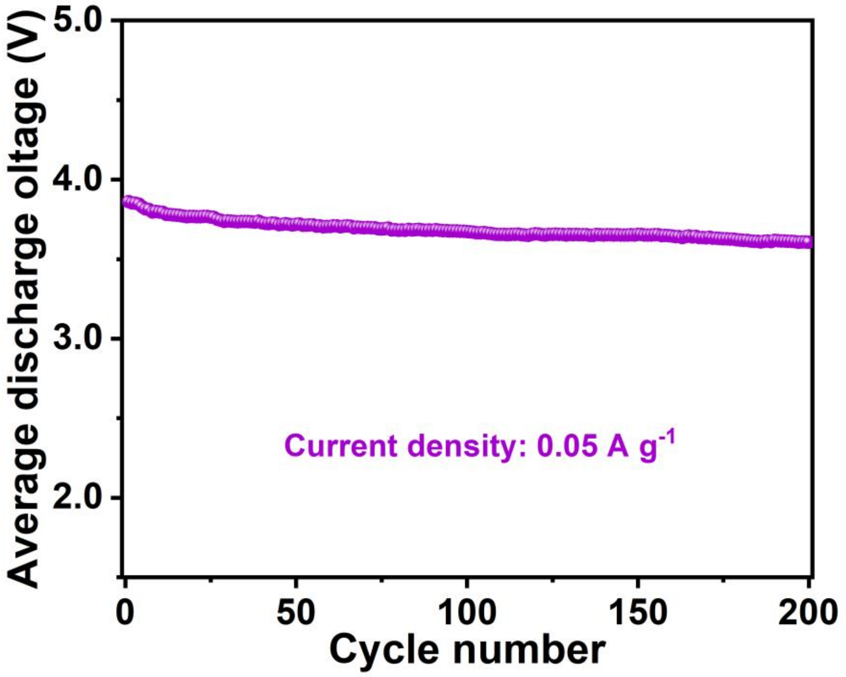

Fig. S33 Average discharge voltage of the SDIB at 0.05 A g^{-1} over 200 cycles

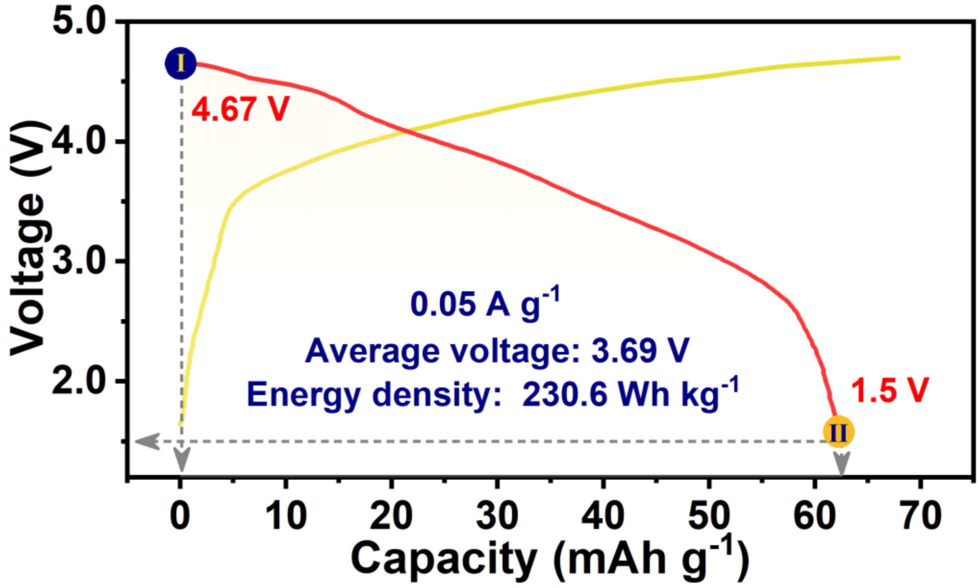

Fig. S34 Galvanostatic charge/discharge curves of NbSSe/NC//EG SDIBs in the fifth cycle at 0.05 A g^{-1}

Table S2 Comparison of electrochemical performances of anodes for Na-DIBs (the capacity is calculated based on the mass of cathode)

Branches	Materials	Current (mA g^{-1}) /Cycle number	Capacity (mAh g^{-1})	Average discharge voltage (V)	m_a/m_c	Refs.
Carbon materials	Soft carbon‖graphite	333/800	18	3.5	1.5/2.5-3.0	(S1)
	Porous N-doped CNF‖graphite	28.5/346	18.6	4.4	0.5/3-4	(S2)
	P-doped hollow carbon‖EG	125/1500	30.25	3.5	1/4	(S3)
	P-doped soft carbon‖graphite	166.6/100	36.6	3.5	1/3	(S4)
		333/900	27			
	Soft carbon nanosheets‖EG	100/350	56.3	4.2	1.5/2	(S5)
Alloy	Phosphorus‖EG	50/140	26.5	4,1	1/10	(S6)
Metal-oxides	TiO_2‖Graphite	500/1400	33.8	3.2	1/3	(S7)
	$Na_2Ti_3O_7$‖Coronene	111/5000	17.3	2.4	0.9/4.5	(S8)
Metal-sulfides	N,S-doped C@MoS_2 nanosheets‖EG	74.6/300	44.7	2.9	1.5/2	(S9)
		746/5000	29.3			
	$MoS_{1.5}Te_{0.5}$@C nanocables‖EG	250/1500	36.4	3.31	1/4	(S10)
	MoS_2‖Graphite	600/5000	11.5	2.75	1.5/6	(S11)
	WS_2/C@CNTs‖EG	250/500	34.2	3.4	1/4	(S12)
	NbSSe/NC‖EG (This work)	50/100	62	3.69	1/4	/
		500/1000	57	3.45		

Table S3 Comparison of the rate performance of the NbSSe/NC//EG full-cell with recent reported SDIBs

Materials	Current density (mA g^{-1})	Cycle number	Capacity retention (%)	References
NbSSe/NC//graphite	1000	1000	89.1	This work
WSSe@CPCS//graphite	1000	700	70.9	(S13)
Hard carbon//graphite	200	1000	83	(S14)
C@$MoS_{2-x}Te_x$@C//graphite	1000	350	98.9	(S15)
$TiSe_2$//graphite	100	200	83.5	(S16)
(MoS2/CF)@MoS2@C//graphite	125	500	86.1	(S17)
Soft carbon//EG	100	350	94	(S18)
Soft carbon//EG	333	800	81.8	(S1)
Hard carbon//graphite	250	200	71	(S19)

Supplementary References

[S1] L. Fan, Q. Liu, S.H. Chen, Z. Xu, B.G. Lu, Soft carbon as anode for high-performance sodium-based dual ion full battery. Adv. Energy Mater. **7**, 1602778 (2017). https://doi.org/10.1002/aenm.201602778

[S2] M. Zhang, M. Shoaib, H. Fei, T. Wang, J. Zhong et al., Hierarchically porous N-doped carbon fibers as a free-standing anode for high-capacity potassium-based dual-ion battery. Adv. Energy Mater. **9**, 1901663 (2019). https://doi.org/10.1002/aenm.201901663

[S3] X. Wang, M. Hou, Z. Shi, X. Liu, I. Mizota et al., Regulate phosphorus configuration in High P-doped hard carbon as a superanode for sodium storage. ACS Appl. Mater. Interfaces **13**, 12059-12068 (2021). https://doi.org/10.1021/acsami.0c23165

[S4] R. Ma, L. Fan, S. Chen, Z. Wei, Y. Yang et al., Offset initial sodium loss to improve coulombic efficiency and stability of sodium dual-ion batteries. ACS Appl. Mater. Interfaces **10**, 15751-15759 (2018).

https://doi.org/10.1021/acsami.8b03648

[S5] X. Yao, Y. Ke, W. Ren, X. Wang, F. Xiong et al., Defect-rich soft carbon porous nanosheets for fast and high-capacity sodium-ion storage. Adv. Energy Mater. **9**, 1803260 (2018). https://doi.org/10.1002/aenm.201803260

[S6] D. Yu, L. Cheng, M. Chen, J. Wang, W. Zhou et al., High-performance phosphorus-graphite dual-ion battery. ACS Appl. Mater. Interfaces **11**, 45755-45762 (2019). https://doi.org/10.1021/acsami.9b16819

[S7] X. Wang, L. Qi, H. Wang, Anatase TiO_2 as a Na^+-storage anode active material for dual-ion batteries. ACS Appl. Mater. Interfaces **11**, 30453-30459 (2019). https://doi.org/10.1021/acsami.9b09703

[S8] S. Dong, Z. Li, I.A. Rodríguez-Pérez, H. Jiang, J. Lu et al., A novel coronene//$Na_2Ti_3O_7$ dual-ion battery. Nano Energy **40**, 233-239 (2017). https://doi.org/10.1016/j.nanoen.2017.08.022

[S9] Y. Liu, X. Hu, G.B. Zhong, J.X. Chen, H.B. Zhan et al., Layer-by-layer stacked nanohybrids of N,S-co-doped carbon film modified atomic MoS_2 nanosheets for advanced sodium dual-ion batteries. J. Mater. Chem. A **7**, 24271-24280 (2019). https://doi.org/10.1039/c9ta09636a

[S10] Y. Liu, X. Hu, J. Li, G. Zhong, J. Yuan et al., Carbon-coated $MoS_{1.5}Te_{0.5}$ nanocables for efficient sodium-ion storage in non-aqueous dual-ion Batteries. Nat. Commun. **13**, 663 (2022). https://doi.org/10.1038/s41467-022-28176-0

[S11] J. Fan, Q. Xiao, Y. Fang, L. Li, W. Feng et al., Reversible intercalation of 1-ethyl-3-methylimidazolium cations into MoS_2 from a pure ionic liquid electrolyte for dual-ion cells. ChemElectroChem **6**, 676-683 (2018). https://doi.org/10.1002/celc.201801583

[S12] Y. Liu, J. Li, B. Liu, Y. Chen, Y. Wu et al., Confined WS_2 nanosheets tubular nanohybrid as high-kinetic and durable anode for sodium-based dual ion batteries. ChemSusChem **16**, e202201200 (2022). https://doi.org/10.1002/cssc.202201200

[S13] H. Lei, H. Wang, B. Cheng, F. Zhang, X. Liu et al., Anion-vacancy modified WSSe nanosheets on 3D cross-networked porous carbon skeleton for non-aqueous sodium-based dual-ion storage. Small e2206340 (2022). https://doi.org/10.1002/smll.202206340

[S14] W. Li, Y. Li, X. Liu, Z. Gu, H. Liang et al., All-climate and ultrastable dual-ion batteries with long life achieved via synergistic enhancement of cathode and anode interfaces. Adv. Funct. Mater. **32**, 2201038 (2022). https://doi.org/10.1002/adfm.202201038

[S15] Zong, F. Wang, H. Liu, M. Zhao, S. Yang, Te-doping induced C@$MoS_{2-x}Te_x$@C

nanocomposites with improved electronic structure as high-performance anode for sodium-based dual-ion batteries. J. Power Sources **535**, 231462 (2022). https://doi.org/10.1016/j.jpowsour.2022.231462

[S16] R. Zheng, H. Yu, X. Zhang, Y. Ding, M. Xia et al., A novel $TiSe_2$-graphite dual ion battery: fast Na-ion insertion and excellent stability. Angew. Chem. Int. Ed. **60**, 18430-18437 (2021). https://doi.org/10.1002/anie.202105439

[S17] C. Cui, Z. Wei, J. Xu, Y. Zhang, S. Liu et al., Three-dimensional carbon frameworks enabling MoS_2 as anode for dual ion batteries with superior sodium storage properties. Energy Storage Mater. **15**, 22-30 (2018). https://doi.org/10.1016/j.ensm.2018.03.011

[S18] X. Yao, Y. Ke, W. Ren, X. Wang, F. Xiong et al., Defect-rich soft carbon porous nanosheets for fast and high-capacity sodium-ion storage. Adv. Energy Mater. **9**, 1803260 (2018). https://doi.org/10.1002/aenm.201803260

[S19] X. Jiang, X. Liu, Z. Zeng, L. Xiao, X. Ai et al., A nonflammable Na^+-based dual-carbon battery with low-cost, high voltage, and long cycle life. Adv. Energy Mater. **8**, 1802176 (2018). https://doi.org/10.1002/aenm.201802176
